# Photosensitive tyrosine analogues unravel site-dependent phosphorylation in TrkA initiated MAPK/ERK signaling

**DOI:** 10.1038/s42003-020-01396-0

**Published:** 2020-11-25

**Authors:** Shu Zhao, Jia Shi, Guohua Yu, Dali Li, Meng Wang, Chonggang Yuan, Huihui Zhou, Amirabbas Parizadeh, Zhenlin Li, Min-Xin Guan, Shixin Ye

**Affiliations:** 1 Shanghai Key Laboratory of Brain Functional Genomics, Ministry of Education, East China Normal University (ECNU), Shanghai, 200062 China; 2 Shanghai Key Laboratory of Regulatory Biology, Institute of Biomedical Sciences and School of Life Sciences, East China Normal University (ECNU), Shanghai, 200062 China; 3 Department of Anesthesiology, State Key Laboratory of Cardiovascular Disease, Fuwai Hospital, National Center for Cardiovascular Diseases, Chinese Academy of Medical Sciences & Peking Union Medical College, Beijing, 100037 China; 4 Division of Medical Genetics and Genomics, the Children’s Hospital, Institute of Genetics, Zhejiang University School of Medicine, Hangzhou, Zhejiang, China; 5 Brain Cognition and Brain Disease Institute (BCBDI), Shenzhen-Hong Kong Institute of Brain Science-Shenzhen Fundamental Research Institutions, Guangdong Provincial Key Laboratory of Brain Connectome and Behavior, CAS Key Laboratory of Brain Connectome and Manipulation, Shenzhen Institutes of Advanced Technology, Chinese Academy of Sciences, Shenzhen, 518055 China; 6 UMR 7238 (LCQB unit), Centre National de la Recherche Scientifique (CNRS), Institut National de la Sante et de la Recherche Medicale (INSERM), Institute of Biology, Paris-Seine (IBPS), Sorbonne University, 75005 Paris, France; 7 UMR 8256 (B2A unit), Centre National de la Recherche Scientifique (CNRS), Institut National de la Sante et de la Recherche Medicale (INSERM), Institute of Biology, Paris-Seine (IBPS), Sorbonne University, 75005 Paris, France; 8 Present Address: INSERM U1195 unit, University of Paris-Saclay, 94276 Le Kremlin Bicêtre, France

**Keywords:** Kinases, Phosphorylation

## Abstract

Tyrosine kinase A (TrkA) is a membrane receptor which, upon ligand binding, activates several pathways including MAPK/ERK signaling, implicated in a spectrum of human pathologies; thus, TrkA is an emerging therapeutic target in treatment of neuronal diseases and cancer. However, mechanistic insights into TrKA signaling are lacking due to lack of site-dependent phosphorylation control. Here we engineer two light-sensitive tyrosine analogues, namely *p*-azido-L-phenylalanine (AzF) and the caged-tyrosine (ONB), through amber codon suppression to optically manipulate the phosphorylation state of individual intracellular tyrosines in TrkA. We identify TrkA-AzF and ONB mutants, which can activate the ERK pathway in the absence of NGF ligand binding through light control. Our results not only reveal how TrkA site-dependent phosphorylation controls the defined signaling process, but also extend the genetic code expansion technology to enable regulation of receptor-type kinase activation by optical control at the precision of a single phosphorylation site. It paves the way for comprehensive analysis of kinase-associated pathways as well as screening of compounds intervening in a site-directed phosphorylation pathway for targeted therapy.

## Abstract (web summary)

Using genetic code expansion, Zhao, Shi et al. generate light-sensitive tyrosine analogues to obtain insights into the activation of the NGF receptor, TrkA. They identify light-sensitive and NGF-insensitive phosphorylation sites, validating the approach and providing insights into TrkA signaling

## Introduction

Introducing light-sensitive moieties into proteins provides a powerful approach to investigate molecular mechanisms, as well as biological functions with high-temporal and spatial resolutions^1,2^. An attractive strategy to engineer light responsiveness relies on photoreactive unnatural amino acids (Uaas), allowing site-specific incorporation in a protein target. Uaas with unique light-sensitivity have been successfully incorporated into membrane receptors, GPCRs and ion channels, contributing to our understanding of receptor function^3,4^. However, this approach has not yet been applied to the kinase receptors. Here, we present a light-sensitive tyrosine kinase A (TrkA) design through the genetic incorporation of photoreactive Uaas.

Kinase phosphorylation regulates diverse biological processes in eukaryotic systems^5,6^ and has implications in various diseases, including neuronal pathologies and cancer^7^. Over 500 protein kinases have been identified in humans, producing a plethora of phosphorylation sites on tyrosine, threonine, and serine residues. Each kinase family has a distinct mechanism for the activation and propagation of signals^6^. Up to now, detailed maps and atlases documenting thousands of phosphorylation sites. However, the complete description of modification at each phosphorylation site under any condition has certainly not been elucidated. One of the reasons that protein phosphorylation has been studied so extensively is the fact that Trk receptor family (including TrkA, TrkB, and TrkC) plays a central role in a very broad range of cellular responses and is implicated in a spectrum of human pathologies, including Alzheimer’s, Parkinson’s diseases, pain, and notably cancer^8–13^. Here, we have utilized the TrkA of neuronal growth factor (NGF) as a model membrane kinase to investigate the site-dependent signaling transduction process. The Trk kinase family is expressed in a host of cells throughout the mammalian body, including neurons^8,9,13,14^. TrkA in particular was first to be identified as a specific receptor for NGF and is activated upon NGF binding^15,16^. Activation of TrkA upon NGF binding at the cell surface leads to phosphorylation of tyrosine residues in the cytoplasmic domain, which in turn recruits signaling molecules and activates multiple signaling pathways involving AKT, MAPK/ERK, and PI3K^13^. Single-molecule tracking shows that NGF binding induces TrkA dimerization or oligomerization^17^ to facilitate the recruitment of signaling transduction.

The cytoplasmic region of monomeric TrkA consists of five conserved tyrosine residues Y670, Y674 and Y675 within the tyrosine kinase domain, and Y490 and Y785 outside the kinase domain. Phosphorylation of Y490 requires the recruitment of adaptors Shc or Frs2 for activation of the AKT and MAPK/ERK pathways^13,18^ while phosphorylated Y785 recruits PLC-γ1^13,18,19^. The MAPK/ERK pathways activated by TrkA include ERK1/ERK2, and ERK5, which phosphorylate and activate downstream transcription factors to regulate target gene expression contributing to neuronal differentiation^20^. Phosphorylation on Y670, Y674, and Y675 are less well understood. Based on the conventional mutagenesis, it has been shown that phosphorylation in the activation loop of the kinase domain on Y670, Y674, and Y675 enhances the kinase activity^21^. However, a specific pathway after phosphorylation for each site remains unaddressed.

To understand the pathway, it requires a strategy to control the phosphorylation state of the individual tyrosine with high-spatial and temporal resolution. We have focused on the applications of genetic code expansion technique to reprogram proteins in response to light^22,23^. A significant advance has been made through the development of genetic code expansion involving the readthrough of an amber (UAG) codon in an mRNA by a suppressor tRNA aminoacylated with the unnatural amino acid (Uaa)^24,25^. Numerous Uaas with chemical, physical, and biological properties not present in the natural amino acids have been incorporated into proteins in eukaryotic systems, particularly the mammalian systems by the introduction of engineered aminoacyl-tRNA synthetase (aaRS)/suppressor tRNA pairs that aminoacylate the suppressor tRNA in vivo with the desired Uaa. The light-sensitive Uaas have been demonstrated to switch protein function after light stimulation^23,26^. Light is a unique signal input with three major advantages for studying biological functions: (1) it has high-temporal and spatial resolution to control a biological reaction; (2) reaction kinetics are faster and more specific than small molecule-based controls that rely on molecule diffusion; (3) cooperative mechanisms can be elucidated by combining controls by light and other molecules including ligand^27^.

Our approach takes advantage of the recent development of the genetic code expansion in mammalian cells. We were mainly focused on the introduction of a light-switch to regulate the phosphorylation state of the tyrosine side chain in TrkA. Two types of ultraviolet (UV) light-sensitive tyrosine analogs have been exploited: (1) caged-tyrosine (ONB), which releases a photolabile moiety from the side chain upon light activation^28,29^; (2) photo-cross-linking *p*-azido-L-phenylalanine (AzF), which crosslinks to a nearby molecule within the distance of 3–4 Å upon light activation^30^. The purposes of the present study were several-fold. The first was to devise a suitable light-controlled methodology for the concept that the phosphorylation state of tyrosine can be specifically turned “on” to identify the downstream signaling process involved with the ligand binding in a kinase receptor. The second was, in the case of the TrkA receptor, to determine how three tyrosine residues in the tyrosine kinase domain initiate ERK signaling. We also sought to exploit our biological screens of light-induced ERK function to identify TrkA receptor that can be switched on without the ligand NGF. Finally, we have applied our approach to neuronal SH-SY5Y cell lines, providing evidence for the transferability of light-sensitive TrkA to control neurite growth. By embedding the methodology into the fast-growing field of molecular optogenetics, we highlight both the potential and limitations of this technique, and provide future perspectives for its use in neurophysiology.

## Results

### ONB and AzF site-specific incorporation into TrkA-Y490

**Fig. 1:**
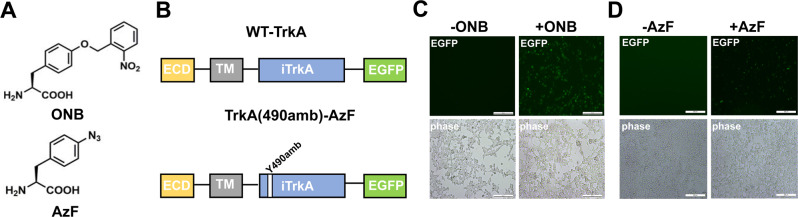
The ONB and AzF incorporation in TrkA. **a** Structure of ONB and AzF. **b** Schematic diagram of the constructed TrkA receptor tagged with EGFP at the C-term. The full-length wt-TrkA receptor consists of an extracellular domain (ECD), a transmembrane domain (TM), an intracellular domain (iTrkA) and EGFP. TrkA(Y490amb) has an amber mutation (UAG) introduced into the Y490 position in the iTrkA domain. **c**, **d** Fluorescent images of live cells co-transfected with RS/tRNA_4x_ at one site out of tyrosine kinase domain Y490, in the presence (+) or absence (−) of 1 mM AzF (**c**) and 1 mM ONB (**d**).

To establish the site-specific incorporation of ONB and AzF (Fig. 1a) into TrkA, we first constructed EGFP tagged TrkA receptor (wt) and the amber codon mutated TrkA receptor (Y490amb) (Fig. 1b), to detect their expression in mammalian cells using the fluorescent signal. Based on this wt version, the amber mutation (UAG) was introduced into the Y490 position to generate the TrkA(490amb). When co-expressed the TrkA(490amb) with the plasmid encoding pONBYRS/U6-PyltRNA^29^ orthogonal to ONB (Fig. 1c) or the plasmid encoding AzF-RS/Bst-tRNA_CUA_^31^ orthogonal to AzF (Fig. 1d) in the presence of 1 mM Uaa in the cell media, fluorescent signals can be observed, suggesting successful incorporation of ONB or AzF in each condition. This AzF-RS/Bst-tRNA_CUA_ construct was compared with plasmids separately encoding *Ecoli*AzF-RS and Bst-tRNA_CUA_^Tyr^, showing similar expression levels quantified by the dual EGFP-mCherry reporter^31^ (Supplementary Fig. 1). In the absence of AzF, there is a minimal level of fluorescent signal, suggesting the *Ecoli*AzF-RS and Bst-tRNA_CUA_^Tyr^ is orthogonal both in HEK293T and PC12 cells with a minimal level of misincorporation in the stop codon.

To quantify the expression level of TrkA-EGFP, we first established the quantification procedure using a regular soluble EGFPamb reporter construct^31^, which only gives full-length expression protein when the amber mutation at position Y39 is suppressed (Supplementary Fig. 2a), with 44% of cells co-transfected with EGFPamb and AzF-RS/Bst-tRNA_CUA_ showing fluorescent when adding AzF, compared to 100% of cells transfected with wt EGFP. In TrkA(490amb)-EGFP, there was 52.6% of cells showing fluorescent when adding AzF, compared to 100% of cells transfected with TrkA-EGFP (Fig. 1d and Supplementary Table 1), indicating that a remarkable yield of the membrane receptor TrkA-EGFP, which is comparable to the water-soluble EGFP.

### Use HEK293T cells for TrkA site-dependent ERK activation

**Fig. 2:**
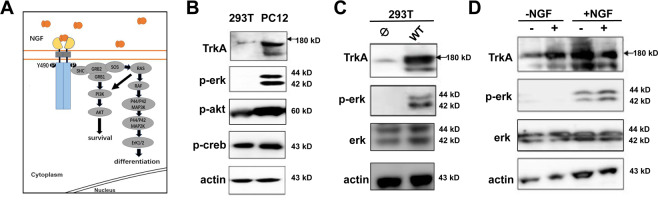
ERK activation by TrkA. **a** Schematic diagram of TrkA signaling pathway. **b** Western blot analysis of PC12 and HEK293T cells due to endogenous TrkA in the presence of ligand NGF for p-erk (phosphorylated ERK at Thr202 and Tyr204), phosphorylated akt, phosphorylated CREB. β-actin was used as loading control. **c** Western blot analysis of HEK293T cells in non-transfected (Ø) and transfected with wt-TrkA (wt) probing for endogenous TrkA, p-erk and total ERK. β-actin was used as loading control. **d** Western blot analysis of HEK293T cells expressing wt-TrkA, without (−) or with (+) NGF, and without (−) or with (+) UV light, for pERK. β-actin was used as loading control.

NGF binding to TrkA induces AKT or MAPK/ERK signaling cascades, and the Y490 site has been associated with these two pathways inferred by the mass-spectrometry analysis^18^ (Fig. 2a). To determine the TrkA dependent pathway activations, we probed the endogenous TrkA activation of MAPK/ERK in HEK293T and PC12 cell line. HEK293 cells are popular cell hosts for gene expression and analysis of the expressed protein due to its transferability by the various techniques. In comparison, the PC12 cells are useful as a model system for NGF neuronal differentiation and neurosecretion. We determined MAPK/ERK activation by the immunoblot analysis of phosphorylated ERK (p-ERK1/2, Thr202 and Tyr204), phosphorylated Akt and phosphorylated creb (p-creb, Ser133) (Fig. 2b). In both cases, HEK293T cells and PC12 cells were treated with NGF for 10 minutes. Following ligand application, cells were immediately lysed and probed for TrkA, p-erk, p-akt, p-creb and actin as a loading control. In HEK293 cells where little endogenous TrkA was detected (with a faint band observed), there is no detectable p-erk. In contrast, PC12 cells showed a high level of TrkA expression, it showed strong p-erk. For both cells, similar levels of p-creb and p-Akt were observed. HEK293T cells transfected with TrkA demonstrated p-erk signal, demonstrating a clear contrast from the non-transfected cells (Fig. 2c).

To directly compare the expression of Uaa incorporated TrkA in HEK293T or PC12 cells, we co-transfected the low-differentiating PC12 cells using the same protocol as in HEK293T cells keeping the TrkA-Y490amb and AzF-RS/Bst-tRNA_CUA_ at same gene dosages. We observed that the yield in PC12 cells is significantly lower (3% of the WT-TrkA) (Supplementary Figs. 3 and 4) than in HEK293 cells (52.6% of the WT-TrkA) presumably due to the challenge in lipofectamine mediated transient transfection in the neuronal cell line. We further characterized TrkA-Y490AzF induced ERK activation in HEK293 cells (Fig. 2). The expression of TrkA-Y490AzF in HEK293T cells was analyzed by fluorescent imaging and western blotting using wild-type TrkA as a control (Supplementary Fig. 5), which showed general agreement. TrkA-Y490AzF was synthesized only in those cells co-expressing *Ecoli*AzF-RS and Bst-tRNA_CUA_^Tyr^ in the presence of exogenously added AzF (Supplementary Fig. 5a–c, lane 4). In the absence of AzF, we observed no expression of TrkA by western blotting analysis (Supplementary Fig. 5a–c, lane 3) and only negligible EGFP signal (Supplementary Fig. 5a, b, lanes 3), confirming the stop of translation. The measurement of EGFP signals allowed us to quantitate the level of TrkA-Y490AzF expression, which was consistently found to be 52.6% of wild-type TrkA expression levels. Thus, we made the use of EGFP to visualize the expression of TrkA. We then studied the p-erk activation of TrkA-Y490AzF. Interestingly, immunoblot analysis of p-ERK showed that TrkA-Y490AzF mutant could not initiate p-erk (Supplementary Fig. 5c), revealing the replacement of azido group at Y490 site inhibited the autophosphorylation. In summary, these results showed that HEK293T is a convenient cellular model compared to PC12 cells to express TrkA site-specifically incorporated with a UAA and study the MAPK/ERK signaling. Unlike PC12 with a high level of endogenous TrkA expression, HEK293T has a low level of endogenous TrkA, it exhibits clear-cut p-erk activation only when exogenous TrkA was expressed.

### Incorporation of AzF and ONB in tyrosine kinase domain sites

**Fig. 3:**
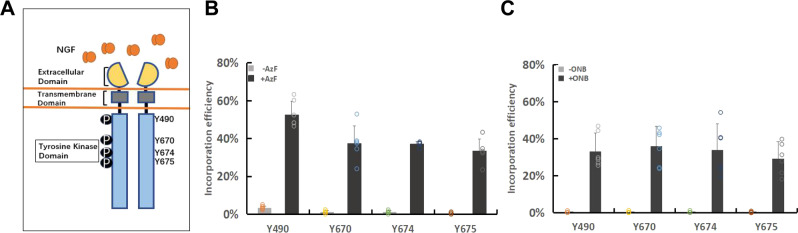
Expression of TrkA mutants containing AzF or ONB at positions 490, 670, 674, and 675, respectively. **a** Schematic diagram of the full-length TrkA with ECD, TM and intracellular TrkA domains. Ligand NGF binds at the ECD domain. Four phosphorylated tyrosine sites subjected to mutation are highlighted. **b** The incorporation efficiency of TrkA AzF mutants in the absence (gray) or presence (dark) of AzF. **c** The incorporation efficiency expression of TrkA ONB mutants in the absence (gray) or presence (dark) of ONB. The incorporation efficiency was described as the percentage ratio of average fluorescence intensity in cells expressing Uaa-incorporating receptors divided by the average fluorescence intensity in cells expressing the WT receptors. *n* = 6 independent experiments. Error bars show s.d.

Using the Y490 site as a reference, we next studied the incorporation of AzF and ONB into TrkA at three different sites in the tyrosine kinase domain, Y670, Y674, and Y675, respectively (Fig. 3a). Fluorescent imaging analysis showed that TrkA-AzF mutants were produced to ~50% (ranged from 33.4 to 52.6%) of the WT-TrkA (Fig. 3b, Supplementary Fig. 6a, and Supplementary Table 1). In comparison, TrkA-ONB mutants all had a slightly lower level of expression than those containing AzF, ranged from 29.1 to 35.8% of the WT-TrkA (Fig. 3c, Supplementary Fig. 6b, and Supplementary Table 1). The average yields of TrkA mutants were calculated (Supplementary Table 1). The differences in the yields measured by fluorescent imaging overall suggest that we observe efficient amber suppression at various target sites and consequently efficient synthesis of receptor proteins containing AzF and ONB. In addition, WT-TrkA and its mutant variants localized primarily on the plasma membrane (Supplementary Fig. 7a, b).

### UV initiated site-dependent ERK signaling

**Fig. 4:**
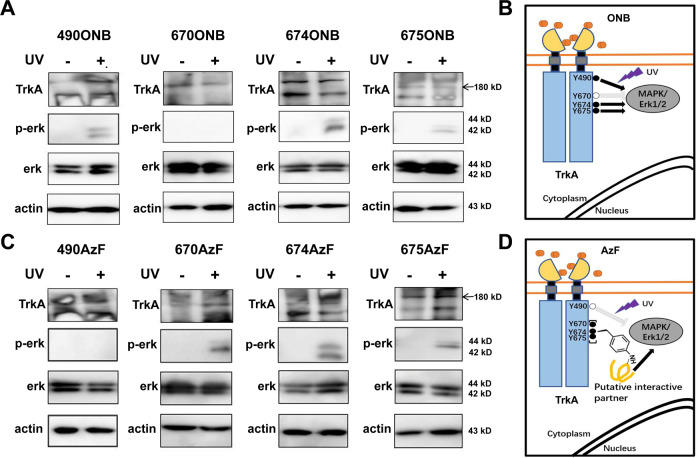
UV light activates MAPK/ERK signaling pathway by site-specifically incorporated AzF and ONB in TrkA. **a** Western blot analysis of HEK293T cells expressing ONB mutants for phosphorylated ERK (Thr202 and Tyr204) in the presence of ligand NGF without (−) or with (+) UV light. **b** Binding of the NGF drives TrkA dimerization, autophosphorylation and initiates intracellular MAPK/ERK. In the presence of ligand NGF, ONB incorporated at the Y490, Y674, and Y675 (solid circles) sites in TrkA, respectively, after UV treatment initiate intracellular MAPK/ERK signaling (arrows). ONB incorporated in Y670 (empty circle) site does not initiate intracellular MAPK/ERK signaling (inhibited arrow) **c** Western blot analysis of HEK293T cells expressing AzF mutants for phosphorylated ERK (Thr202 and Tyr204) without (−) or with (+) UV light. UV photolysis was done with 365 nm UV cross-linker CL-1000L (Analytik Jena) at 40w for 5 min at 4 °C. TrkA and β-actin were used as loading control. **d** Binding of the NGF drives TrkA dimerization, autophosphorylation and initiates intracellular MAPK/ERK. In the presence of ligand NGF, AzF incorporated at the Y670, Y674, and Y675 (solid circles) sites in TrkA, respectively, after UV treatment initiate intracellular MAPK/ERK signaling (arrows) presumably due to the crosslinking with a putative interactive protein. AzF incorporated in Y490 (empty circle) site does not initiate intracellular MAPK/ERK signaling (inhibited arrow).

We first tested the UV-stimulated activation of p-erk with the mutant TrkA-Y490ONB. Under UV light stimulation, ONB releases the caged group that restores the tyrosine, and the hydroxyl-group on tyrosine undergo autophosphorylation (Supplementary Fig. 8a and Supplementary Fig. 11a, b). To evaluate whether ONB photo-activation at Y490 site produces functional changes in ERK signaling, we applied UV light on live-cells expressing the mutant and by immunoblot analysis of p-erk post-UV treatment. HEK cells were transfected with the TrkA-Y490ONB and subjected to continuous UV light stimulation (40w) for 5 min in the presence of ligand NGF. Following light illumination, cells were immediately lysed and probed for p-erk, total erk, and actin as a loading control. In the absence of UV light, no p-erk was detected in TrkA-Y490ONB due to the presence of the caged group in ONB. UV illumination induced the clear appearance of p-erk compared to the control TrkA-Y490ONB (Fig. 4a). In summary, our results successfully demonstrated the feasibility of p-erk activation through the light control at a single phosphorylation site in TrkA, in which the Uaa ONB serves as a robust light-switch to control the phosphorylation state.

We then studied whether incorporation of ONB into any of other three tyrosines could also initiate p-erk signaling in TrkA-670, 674, and 675 (Fig. 4a and Supplementary Fig. 11). In the absence of UV treatment, no p-erk was detected for any of the mutants. After UV treatment, two mutants (TrkA-Y674ONB and TrkA-Y675ONB) showed robust p-erk activation similar to what has been observed in the TrkA-Y490ONB mutant. Interestingly, the TrkA-Y670ONB mutant showed no detectable p-erk with or without UV treatment. To quantify the extent of p-erk, the intensity of p-erk signal was measured and normalized to the total ERK signal among all the mutants either in dark or under UV illumination (Supplementary Fig. 9a and Table 1). After UV light stimulation, TrkA-Y490ONB initiated 36.2% of p-erk, TrkA-Y674ONB initiated 44.6% and TrkA-Y675ONB initiated 32.0%, significantly more than their absence of UV controls (16.3%, 7.3%, and 4.4%, respectively) and the light-insensitive mutant TrkA-Y670ONB (absence of UV: 3.1%, presence of UV: 3.6%). As an additional control, we have made conventional mutants namely: TrkA-Y490F, Y670F, Y674F, Y675F, Y490D, Y670D, Y674D, and Y675D. The phenylalanine (F) is considered to abolish the phosphorylation, whereas the aspartic acid (D) may mimic the phosphorylated state. For all the F mutants, we have observed a significant reduction of the p-erk in compared to the wild-type receptors (Supplementary Fig. 10). These results demonstrated that ONB as a light-switch is sufficient for the gain of function to induce a downstream ERK signaling provided with the incorporation at a proper phosphorylation regulatory site (Fig. 4b). Among the three tyrosines (Y670, Y674, and Y675) considered being within the activation loop of the TrkA kinase domain, Y674 and Y675 (but not Y670) may share the same mechanisms as the Y490 to initiate pERK signaling by autophosphorylation.

**Table 1:** All sites were detected under the same conditions as wt, and were repeated more than three times.

Relative level of p-erk (%)(+NGF)	ONB		AzF	
	−UV	+UV	−UV	+UV
(a)				
wt	65.1 ± 0.2	56.8 ± 0.1		
TrkA-Y490	16.3 ± 0.1	36.2 ± 0.2	11.4 ± 0.2	7.8 ± 0.09
TrkA-Y670	3.1 ± 0.004	3.6 ± 0.009	12.4 ± 0.07	34.1 ± 0.09
TrkA-Y674	7.3 ± 0.002	44.6 ± 0.2	12.5 ± 0.03	29.7 ± 0.02
TrkA-Y675	4.4 ± 0.005	32.0 ± 0.1	10.1 ± 0.05	37.4 ± 0.2
(b)				
wt	16.4 ± 0.009	10.4 ± 0.01		
TrkA-Y490	18.5 ± 0.2	16.9 ± 0.2	12.5 ± 0.04	8.6 ± 0.008
TrkA-Y670	9.7 ± 0.07	16.7 ± 0.2	8.4 ± 0.08	17.6 ± 0.2
TrkA-Y674	10.6 ± 0.08	34.5 ± 0.1	26.6 ± 0.1	80.4 ± 0.3
TrkA-Y675	3.7 ± 0.01	37.1 ± 0.2	7.9 ± 0.05	39.0 ± 0.2

Table 1(a) list all conditions treated with NGF (+NGF) and 1(b) without UV treatment (−NGF). For each measurement, the intensity of phosphorylated ERK to total ERK was measured, and the ratio of p-ERK/total ERK was calculated and presented as the relative level of p-erk (%). Error show s.d.

We next examined among all four sites the light-induced effect of AzF, which undergoes different photochemistry than ONB. Upon UV excitation, AzF usually generates a nitrene radical, which may reduce to amine or forms a covalent linkage with the nearby atom of a protein at a distance of 3–4 Å^30,32^ (Supplementary Fig. 8b). Strikingly, except the TrkA-490AzF mutant showing no detectable p-erk after UV light stimulation (absence of UV: 11.4%, presence of UV: 7.8%), all three mutants TrkA-Y670AzF, TrkA-Y674AzF, and TrkA-Y675AzF demonstrated UV-controlled p-erk activation (34.1%, 29.7%, and 37.4%, respectively) (Fig. 4c, Supplementary Fig. 9a, and Table 1), significantly more than their controls treated without UV (12.4%, 12.5%, and 10.1%, respectively). Interestingly, the Y674D and Y675D mutants also demonstrated different levels of reduction of p-erk. The Y490D and Y670D mutants maintained the same level of pERK activation compared to the WT. Taken together, these results indicate that AzF can serve as a light-switch to activate downstream ERK signaling in TrkA receptors, presumably through the AzF mediated photo-cross-linking mechanism (Fig. 4d) that is different from the photochemistry, which generated phenylamino side chain (Supplementary Fig. 8a, b).

### NGF independent light-controlled ERK signaling

**Fig. 5:**
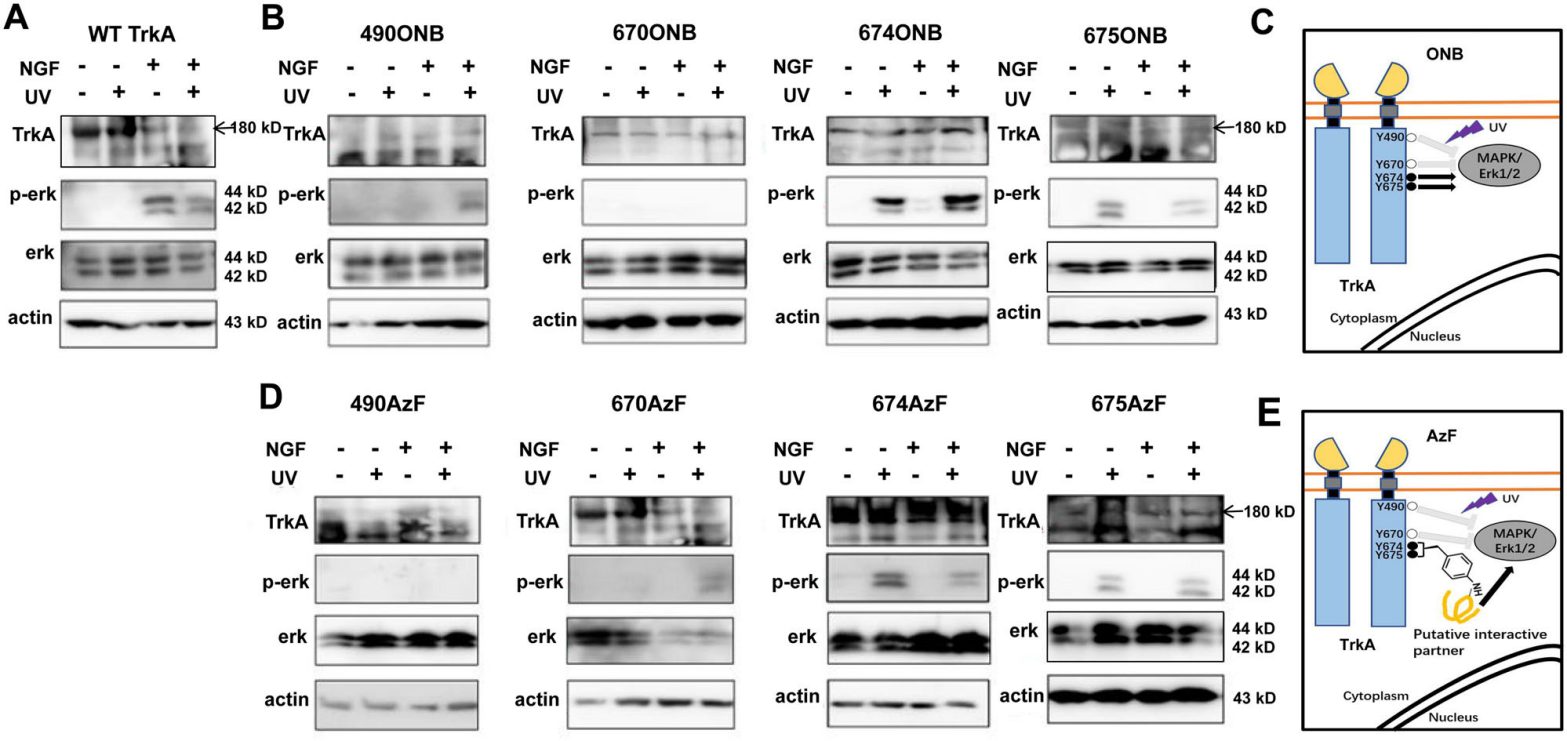
UV light activates MAPK/ERK signaling pathway independent of ligand NGF. Western blot analysis of HEK293T cells expressing wt-TrkA (**a**), ONB mutants (**b**) including TrkA-Y490ONB, TrkA-Y670ONB, TrkA-Y674ONB, TrkA-Y675ONB, and AzF mutants (**d**) including TrkA-Y490AzF, TrkA-Y670AzF, TrkA-Y674AzF, TrkA-Y675AzF; without (−) or with (+) NGF, and without (−) or with (+) UV light. TrkA and β-actin were used as loading control. In the absence of ligand NGF, ONB (**c**) or AzF (**e**) incorporated at the Y674 and Y675 (solid circles) sites in TrkA, respectively, after UV treatment initiate intracellular MAPK/ERK signaling (arrows). In the case of AzF, the signaling activation is presumably due to photo-cross-linking of an interactive protein in the MAPK/ERK signaling complex. ONB (**c**) or AzF (**e**) incorporated in Y490 and Y670 (empty circle) site does not initiate intracellular MAPK/ERK signaling (inhibited arrow).

Next, we tested whether UV light stimulation in TrkA could directly activate ERK without NGF (Fig. 5a and Supplementary Fig. 11j–m). All mutants were expressed and assayed in the absence and presence of NGF, and wt-TrkA serves as a control with no detectable p-erk neither without nor with UV treatment, confirming that the UV treatment per se does not cause significant functional changes. Interestingly, among four ONB mutants, in the absence of NGF, both TrkA-Y674ONB and TrkA-Y675ONB showed UV-induced p-erk activation compared to the control without UV treatment (80.4% and 39.0%, respectively) (Fig. 5b, Supplementary Fig. 9b, and Table 1). The UV dependent activation was similar to the controls treated in the presence of NGF. In contrast, the TrkA-Y490ONB only demonstrated UV light-induced p-erk activation in the presence of NGF, whereas the TrkA-Y670ONB showed no detectable p-erk activation under all conditions (Fig. 5b, c). We repeated the same experiment with AzF mutants, similar to the TrkA-Y674ONB and TrkA-Y675ONB, TrkA-Y674AzF and TrkA-Y675AzF had NGF independent UV-induced p-ERK activation (34.5% and 37.1%, respectively) (Fig. 5d, Supplementary Fig. 9b, and Table 1). Whereas the TrkA-Y490AzF showed no detectable p-erk activation under all conditions, and the TrkA-Y670AzF only demonstrated UV light-induced p-ERK activation in the presence of NGF. All these results validated that light-induced activation through AzF or ONB incorporation of p-erk is highly effective in mimicking NGF function at Y674 and Y675 sites (Fig. 5d, e).

To confirm if TrkA AzF mutant crosslinks to some proteins under UV treatment, we implement the photo-cross-linking experiment. We tested WT-TrkA, TrkA-Y670AzF, TrkA-Y674AzF and TrkA-Y675AzF in the presence of NGF and absence of NGF, without and with UV (Supplementary Fig. 12). For all conditions, TrkA monomer could be detected as a characteristic 180 kDa band. Upon UV exposure, we observed higher molecular bands for TrkA-Y674AzF and TrkA-Y675AzF mutants (~300 kDa) in both +NGF and –NGF conditions, but not in WT or TrkA-Y670AzF.

### Expressing light-sensitive TrkA in neuronal SH-SY5Y cell line and controlling neuronal differentiation

**Fig. 6:**
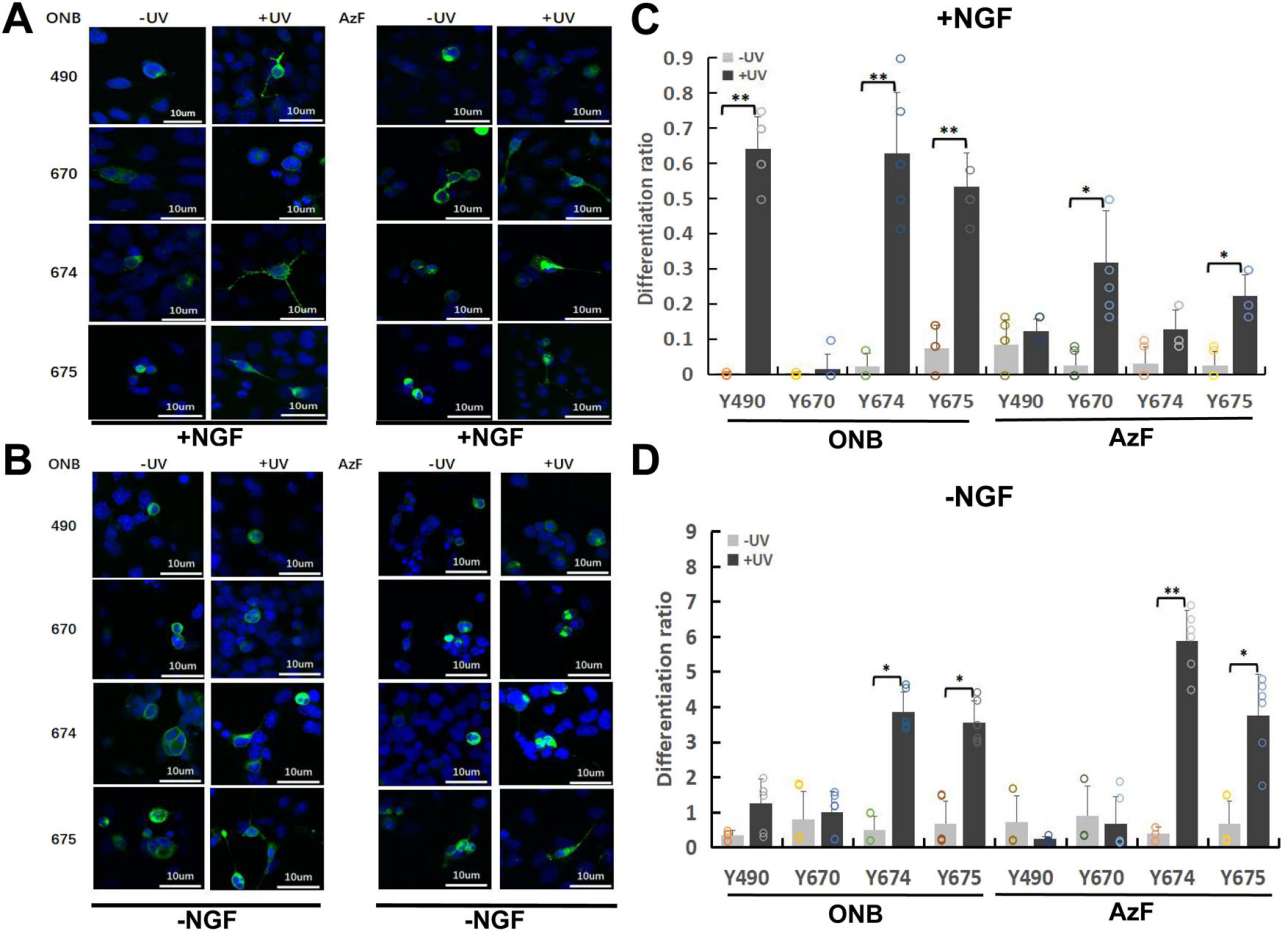
TrkA-ONB and AzF mutants promote SH-SY5Y cell differentiation in response to UV Stimulation with and without NGF. **a** Representative fluorescence images depicting SH-SY5Y cells expressing TrkA-ONB (two columns on the left) or AzF (two columns on the right) mutants incorporated at indicated sites before (-UV) and after (+UV) treatment, with medium supplied with 100 ng/mL NGF for 30 min and kept in the dark for 24 h prior to imaging. **b** Representative fluorescence images depicting SH-SY5Y cells expressing TrkA-ONB (two columns on the left) or AzF (two columns on the right) mutants incorporated at indicated sites before (-UV) and after (+UV) treatment with medium supplied without NGF. **c** Differentiation ratios were calculated for various mutants expressed in SH-SY5Y cells in the presence of NGF with or without UV light. **d** Differentiation ratios calculated for SH-SY5Y cells in the absence of NGF expressing wt-TrkA (WT) or various mutants with or without UV light. Differentiation ratios were calculated as described in the method. Values represent the mean ± SD of six biological replicates (*n* = 6) with >200 cells counted per replicate. Scale bars, 10 µm.

Finally, we sought to test light-sensitive TrkA mutants in neurons. We decided to first concentrate on the establishment of the neurite growth control by light in SH-SY5Y cells, which do not have the endogenous TrkA that would obscure the functional effect from the mutant TrkA, as well as from the light activation. For that, we have tested whether the TrkA-ONB or AzF mutants could be successfully expressed. Following transient transfections of the constructs and supplementation of the culture media with Uaa (1 mM), treated exactly the same as for HEK293 cells and PC12 cells, we observed a large number of bright green florescent cells (Supplementary Fig. 7b), which were absent in controls where Uaa was not added. This demonstrated proper genetic encoding of the Uaa through the rescue of the amber stop codon by the orthogonal tRNA/aaRS pair. Following this successful implementation of light-sensitive TrkA Uaa mutants in SH-SY5Y cells, we then aimed at assaying the neurite growth upon activation of each tyrosine with UV light and compared their differentiation in the presence and absence of NGF (Fig. 6). SH-SY5Y cells do not project neurites with NGF stimulation. However, when they are expressed with TrkA and treated with NGF, cells project neuritis (Supplementary Fig. 7c), which is a process referred to as differentiation. We proceeded to determine whether TrkA-Uaa mutants could achieve a similar cellular outcome with UV light stimulation.

SH-SY5Y cells expressing wt-TrkA showed robust differentiation when treated with 100 ng/mL NGF, indicating that exogenous TrkA retained ligand sensitivity and signaling activity (Supplementary Fig. 7c). UV light did not significantly alter the differentiation. No differentiation was observed for non-transfected cells. To determine the functional role of Y490, Y670, Y674 and Y675 in differentiation, we transfected SH-SY5Y cells with mutant constructs and illuminated them for 30 min as previously described for western blotting experiments (Fig. 6) and compared their neurite growth to the WT-TrkA. In the presence of NGF, cells expressing Y490ONB, Y674ONB, Y675ONB, Y670AzF and Y675AzF showed significant UV-induced differentiation compared with UV untreated condition (Differentiation ratios: 64.2, 62.8, 53.3, 31.9, and 22.2, respectively) (Fig. 6a, c and Supplementary Table 2). In the absence of NGF, Y674ONB, Y675ONB, Y674AzF and Y675AzF showed UV-induced differentiation compared with UV untreated condition (Differentiation ratios: 3.9, 3.6, 5.9, and 3.8, respectively) (Fig. 6b, d and Supplementary Table 2). Overall, there is a general agreement between ERK activity and cell differentiation.

## Discussion

The common concept of TrkA signaling activation is that the binding of ligand NGF from the extracellular domain promotes homo-oligomerization of TrkA on the cell membrane, which subsequently activates downstream signaling pathways. However, because of its intricate downstream pathway associated with multiple phosphorylation sites within the intracellular segment, the TrkA signaling is not yet fully elucidated. Particularly, the three sites Y670, Y674, and Y675 located in the active region of tyrosine kinase, are closely related to enzyme activation. Previous findings indicated that mutation of any of these three activation loop tyrosines leads to diminished TRK function^21,33^, however, specific signaling process related to MAPK/ERK could not be elucidated due to the lack of site-controllable phosphorylation.

We were particularly interested in developing a framework for light-controlled protein function through site-specific control, which requires the availability of homogeneously expressed proteins with a light-switch installed at a specific site within the protein. In the two past decades, optogenetics based on natural light-sensitive proteins has evolved as a powerful strategy to study cellular functions with the precision of protein-specificity^34^. Despite the power of manipulation of biological functions with cell-type and protein specificity, the regulation of a protein function with site-specificity by light remains a major challenge in the optogenetic approach. Although site-specific cysteine/maleimide labeling has been applied to render protein light-sensitive by attaching photo-switching ligands, the method can not be generalized to control functions associated with post-translation modifications such as phosphorylation. Here our approach by exploiting the genetic code expansion technology^23–25^, in combination with the light-sensitive Uaas and site-directed mutagenesis to optically activate membrane kinase TrkA with the site-precision provides a novel concept and tool-kit to investigate the NGF/TrkA signal some with clear-cut readout.

Our technical advantages over classical phosphomimetic mutations to aspartic acid and glutamic acid are that both ONB and AzF are chemically different from tyrosine so to block phosphorylation, and each undergoes different photo-chemistries. After UV light treatment, ONB releases the caging group to recapitulate the molecular and phenotypic consequences of Tyr. Light-dependent quantitative analysis for “gain-of-function” phenotypes lends strong support to the advantage of light-control of signaling process via Uaa to analyze each tyrosine’s contribution to the specific pathway activation, which conventional mutagenesis cannot afford. The two different Uaas provide unique photochemical mechanisms to control TrkA function by light, an advantage that the light-sensitive protein-based optogenetic approach cannot offer. The remarkable tolerance of all tested sites for ONB and AzF incorporation can be attributed to the orthogonality of aaRS/suppressor tRNA pairs extensively optimized in mammalian systems^22,29,31,35,36^. The extent of incorporation of AzF was found to vary from ~52.6 to 33.4%, compared to ONB varying from 29.1 to 35.8%. We found that the mammalian HEK293T and SH-SY5Y cells are better cellular models than the neuronal PC12 cell line in studying TrkA signaling due to two main reasons: (1) minimal interference from the endogenous TrkA, (2) higher yield in amber suppression by the orthogonal aaRS/suppressor tRNA pairs. Between the two types of tyrosine analogs AzF and ONB introduced into four phosphorylation sites, we identified three categories of mutants: (1) UV-insensitive mutants (TrkA-Y670ONB and TrkA-Y490AzF), (2) UV-sensitive mutants in the presence of ligand NGF (TrkA-Y490ONB and TrkA-Y670AzF), (3) UV-sensitive mutants independent of ligand binding (TrkA-Y674ONB, TrkA-Y675ONB, TrkA-Y674AzF and TrkA-Y675AzF).

Their responses of ERK activation indicate two points of interest: (1) differences of ONB and AzF in Y490 and Y670 sites, and (2) ligand-independency observed for Y674 and Y675 sites. The interpretation of differential light activation between ONB and AzF in site Y490 is the most straightforward. The activation of the ERK pathway by irradiation cannot occur unless the caged compound ONB on Y490 has been released. The difference between Y490ONB being ligand-dependent for light-controlled p-erk activation may reflect the ligand-induced state-dependent activation process. When mutating Y490 to AzF, before light treatment, it is similar to conventional site-directed mutagenesis such as phenylalanine, which leads to the inactivation of MAPK/ERK signaling. The lack of ERK activation after UV treatment confirms that the AzF used different photochemistry to ONB and the photo-product of AzF was unable to be phosphorylated. However, for the site of Y670, surprisingly we have observed that the introduction of AzF resulted in a light-sensitive ERK activation but not in ONB mutation. This represents the first observation that a photo-product of AzF leads to the activation of a signaling pathway. Our interpretation is the following: unlike Y490, the Y670 locates in the activation loop that has been proposed to undergo significant conformational changes upon ligand binding^21^. Although we observed Y670 as a site in which ONB and AzF showed clear differences in light-sensitivity, more direct structural data is required to infer information about the functional difference. It is probable that the nitrobenzyl-caging group in the ONB obstructs the loop conformation and induces an unfavorable conformation for kinase activity both before and after light stimulation. When AzF was introduced, due to the smaller size of the side chain more closely mimicking the tyrosine, such obstruction did not occur. After light stimulation, the activation of ERK signaling suggests that AzF may undergo crosslinking to an interactive counterpart that triggers the ERK signaling cascade (Fig. 5c, e).

We provide here a step in the development of light-controlled kinase receptors in a ligand-independent manner. Our results in Y674 and Y675 sites incorporated with ONB or AzF all demonstrate that the ERK signaling is independent of ligand binding. Previously, it has been shown that adaptor proteins Grb2, SH2B, and rAPS can bind to the phospho-tyrosines at Y670, Y674, and Y675^37,38^. Therefore, our results may suggest all three Y670, Y674, and Y675 sites are using protein–protein interactions to activate MAPK/ERK pathway, presumably through one or more of those adaptor proteins. AzF as a photocrosslinking Uaa has been previously applied in probing protein-ligand^30,31,39,40^ and protein–protein^3,41,42^ interactions. Our crosslinking results on the TrkA-AzF mutants demonstrate the potential of the approach to investigate the light-dependent signaling mechanisms^43,44^, and opens the door for us to investigate the putative binding partners of the TrkA receptors. By coupling with mass-spectrometry, we foresee identifying the docking partners of TrkA docking at specific sites. We also note that AzF is a versatile Uaa with multiple applications, including serving as an infrared probe^22^ or chemical handle for bioorthogonal conjugations^45^. This is a unique advantage of the AzF incorporation that other methods cannot afford. Taken together other works on photocaged tyrosines and lysines^29,46,47^ in light-controlling intracellular kinase activity, the availability of multiple Uaas with different chemical properties open new avenues for using genetically encoded Uaas to signaling transduction, an approach likely to extend to other membrane kinase receptors such as epidermal growth factor receptor (EGFR), p75NTR (low-affinity NGF binding receptor), insulin-like growth factor receptor (IGFR), which are all structurally and functionally related to TrkA^13,21^. The genetic encoding technique is also well-suited for biological applications as it allows for both receptor-subtype selectivity and cell-type specificity.

In conclusion, our results demonstrate that Uaas can be efficiently introduced into membrane kinases to render them light-sensitive. The broad applicability of light-sensitive Uaas in molecular engineering and cellular physiology emphasizes its significance in the fields of optogenetic pharmacology and optical monitoring of protein conformational changes. A wealth of structural and functional information can be gained using this technology in vitro on recombinant proteins, while, in vivo, it has the potential to address fundamental questions regarding native receptor biology. Given its broad utility and functionally rich implementation, Uaa insertion is poised to become increasingly popular for investigating protein structure, function and dynamics. By scanning through different phosphorylation sites, we managed to identify light-sensitive TrkA independent of ligand NGF. Compared to the recent demonstration of ligand-independent light-sensitive TrkA based on the fusion of light-sensitive protein Arabidopsis cryptochrome 2^48–50^, our TrkA relies on the light activation of a specific phosphorylation site activating a specific signaling pathway using the two kinds of orthogonal tyrosyl-tRNA and pyrrolysyl- tRNA synthetase/tRNA_CUA_ pairs to incorporate light-sensitive Uaas. The introduction of Uaas to TrkA creates a much smaller change (10-100 Dalton) to the receptor than the light-sensitive proteins (~100 K Dalton); therefore, it is likely to maintain the function of the receptors, including ligand binding, protein–protein interactions and oligomerization. Since both pairs can be used to site-specifically incorporate Uaas in the multicellular model organisms, including zebrafish and mice we^27^ and others developed^51,52^, following our demonstration of a proof-of-concept in neuronal SH-SY5Y cells, we foresee the development of this strategy to photo-control signaling and enzymatic processes in optically targeted single cells in primary neurons and ultimately in living animals, especially by removing the interference of endogenous targeted protein. We envision a comprehensive analysis of Boolean logic with UV light, the phosphorylation site, and the ligand as inputs, to elucidate mechanisms in vivo intervening a site-directed phosphorylation pathway for targeted therapy.

## Methods

### Materials

*p*-azido-L-phenylalanine (AzF) was purchased from Chem-Impex International (Wood Dale, IL) and ONB was synthesized^29^. Standard *E. coli* genetic techniques were performed^53^. *E. coli* strains XL-10 gold strain (Tet^r^∆ (*mcr A)183 ∆(mcr CB-hsdSMR-mrr)173 endA1 supE44 thi-1 recA1 gyrA96 relA1 lac* Hte [F’ *proAB lacI*^*q*^*Z∆M15* Tn*10* (Tet^r^) Amy Cam^r^] from ZEYE and TOP10 [F- *mcrA Δ(mrrr-hsdRMS-mcrBC) φ80lacZΔM15 ΔlacX74 recA1 araD139 Δ(ara-leu)7697 galU galK rpsL (StrR) endA1 nupG*] from Invitrogen were used for plasmid propagation and isolation. Oligonucleotides were obtained from Eurofins and Gene Synthesis Technologies. Plasmid DNA was purified using standard Maxi Prep Kits from Generay.

### Plasmids for expression of EGFP, EGFP-mCherry, and TrkA-EGFP in mammalian cells

The plasmids encoding wild-type EGFP (EGFP.wt), a variant thereof carrying a Y39am mutation (EGFP.Y39am) and EGFP-mCherry carrying an amber mutation were described earlier^31^. Plasmid PLNCX carrying the gene for wild-type rat TrkA has been described^54^. An EGFP insert was prepared by PCR reaction and subcloned into PLNCX-TrkA between the ClaI and StuI sites. This ligation created an inframe fusion between the TrkA carboxyl terminus truncating the last 4 amino acids and the EGFP amino terminus. Amber mutations were introduced into TrkA (TrkA.amb) at positions Y490, Y670, Y674, and Y675 using Quikchange mutagenesis (Stratagene). All plasmid constructs were confirmed by automated DNA sequencing.

### Plasmids for expression of suppressor tRNA and mutant aaRS genes in mammalian cells

Plasmid AzF-RS(tRNA)_4X_ carrying the gene encoding the mutant *E. coli* Tyr tRNA-synthetase for AzF and the amber suppressor tRNA derived from *B. stearothermophilus* Tyr-tRNA_CUA_ has been described previously^31,36^. The plasmid carrying the gene encoding the mutant *Methanosarcina barkeri* pyrrolysine tRNA synthetase for ONB and the amber suppressor tRNA PylRS/tRNA_CUA_ pair constructed as previously described^29^.

### Cell culture, transfection, and incorporation of AzF and ONB

HEK293T cells (ATCC) were maintained in medium containing Dulbecco’s modified Eagle medium (DMEM) (4500 mg/L of glucose; Gibco, Cellgro) supplemented with 10% fetal bovine serum (Biogen). PC12 neuronal cells (ATCC) were maintained in medium containing 1640 (Thermo Fisher), 10% fetal bovine serum (Biogen). SH-SY5Y cells (ATCC) were maintained in medium containing DMEM/F12 (4500 mg/L of glucose; Gibco, Cellgro) supplemented with 10% fetal bovine serum (Biogen) and 1x Penicillin–Streptomycin solution). All cells were cultured at 37 °C in a 5% CO_2_ atmosphere. Cells were washed with phosphate-buffered saline, detached using trypsin/EDTA-solution, re-suspended in fresh growth medium and seeded into cell culture dishes. The cells were routinely tested for mycoplasma contamination. Cells were transfected with plasmid DNA using Lipofectamine 2000 (Invitrogen) according to the manufacturer’s protocol. For transfection in a single well of a 6-well plate, 2 μg of AzF-RS(tRNA)_4X_, 2 μg of PylRS/tRNA_CUA_, 2 μg of TrkA.amb (or EGFPamb, or EGFP-mCherry) were mixed with Lipofectamine 2000 and added to cells at 70–80% confluence according to the manufacturer’s protocol. Four hours after transfection, cells were fed with an equal volume of DMEM supplemented with 10% fetal bovine serum. The unnatural amino acid AzF was supplied at a final concentration of 1 mM four hours post-transfection while o-nitrobenzyl (ONB) was supplied at a final concentration of 50 μM 4 h post-transfection. Cells were harvested 24–48 h post-transfection and fluorescence imaged or analyzed by western blotting. Suppression of the stop codon in EGFP, EGFP-mCherry, or TrkA results in full-length EGFP, whose fluorescence signal reports the incorporation of AzF or ONB at the TAG stop codon. In the presence of 1 mM AzF in the growth media, EGFP fluorescence intensity of cells, as measured by fluorescence microscopy, increased in comparison to the absence of AzF, indicating the incorporation of AzF or ONB into EGFP, EGFP-mCherry or TrkA.

### Microscopy

Forty-eight hours post-transfection, medium was replaced with fresh DMEM and cells analyzed with a Laser-scanning confocal microscope using Bio-Rad Radiance 2100 system mounted on a Nikon Eclipse TE300 inverted microscope equipped with a Plan Fluor EL WD 20x/0.45 objective. Leica DMI 4000B intelligent inverted fluorescence microscope Fluorescence emission of mCherry at above 560 nm was measured using 543 nm excitation. Fluorescence emission of EGFP at above 515–530 nm was measured using 488 nm excitation.

### Western blotting

Cells were washed with PBS, lysed in RIPA buffer (Sigma-Aldrich), resolved by sodium dodecyl sulfate polyacrylamide gel electrophoresis and transferred onto the immunoblot polyvinylidene fluoride membrane (Millipore) according to the protocols provided by the manufacturer. Protein concentration was determined via BCA assay (Thermo Fisher) according to manufacturer’s instructions. The membranes were probed with primary antibodies against the TrkA epitope (Sigma-Aldrich; 1:2000), Myc-tag on TrkA (Abmart; 1:1000), p-Erk1/2 (Millipore; 1:1000), actin (Sigma-Aldrich; 1:1000) and a horseradish peroxidase-conjugated secondary antibody (Bio-Rad; 1:5,000–1:10,000; anti-mouse or anti-rabbit IgG). Signals were visualized by enhanced chemiluminescence treatment (Amersham GE or Perkin Elmer Life Sciences) and exposure to ImageQuant. Equal amounts of total protein were analyzed per lane.

### NGF and UV treatment

Before NGF stimulation and photolysis experiments, medium was replaced with fresh Dulbecco’s phosphate-buffered saline without AzF or ONB, and cells incubated for 2 h at 37 °C in 5% CO_2_ atmosphere. Stimulation was achieved by adding human NGF (Millipore) to a final concentration of 50 ng/ml. As control, an equal amount of NGF-free medium was used. Cells incubated for 15 min, and then they were either washed with ice-cold PBS directly for directly western analysis or for UV treatment. UV photolysis was done with 365 nm UV cross-linker CL-1000L (Analytik Jena) at 40w for 5 min. Afterwards, cells were washed with ice-cold PBS, lysed with RIPA buffer (Sigma) supplemented with phosphatase inhibitors (Pierce) and subjected to western analysis.

### SH-SY5Y cell differentiation assay

Cells were harvested 48 h post-transfection and treated without or with NGF, and without or with UV as mentioned above. Differentiation ratios for each mutant at various conditions were calculated at various conditions: Differentiation ratios = (*N*_m_/*N*_mt_)/(*N*_WT_/*N*_WTt_).

*N*_m_ = Number of green fluorescing cells expressing a specific mutant with neurite length longer than the cell body diameter

*N*_mt_ = Number of total green fluorescing cells expressing a specific mutant

*N*_WT_ = Number of green fluorescing cells expressing WT-TrkA with neurite length longer than the cell body diameter

*N*_WTt_ = Number of total green fluorescing cells expressing WT-TrkA

### Quantification and statistical analysis

Quantification of western blots was carried out using ImageJ. Details of all statistical datasets are included in the corresponding figure legends.

### List of primers

TrkA-Y490ambF: 5′-GGGAGCAAACAGGATTAGATACCCT-3′

TrkA-Y490ambR: 5′-GAGGTTAACAGAGTGACAGATGGTGCA-3′

TrkA-Y670ambF: 5′-GCATGAGCAGGGACATC-3′

TrkA-Y670ambR: 5′-TAGAGCACAGACTACTACCG-3′

TrkA-Y674ambF: 5′-GACTACAGCACAGAC-3′

TrkA-Y674ambR: 5′-TAGTACCGTGTGGGAGGTC-3′

TrkA-Y675ambF: 5′-CTACAGCACAGACTAC-3′

TrkA-Y675ambR: 5′-TAGCGTGTGGGAGGTCGG-3'

### Statistical and reproducibility

All statistical analyses were performed using Microsoft Office Excel. Data are expressed as means ± S.E. Means of two groups were compared using Student′s *t*-test (unpaired, two-tailed), with *P* < 0.05 was considered to be statistically significant. All the experiments were performed at least three times with similar results.

## Supplementary information

Supplementary Information

Description of Additional Supplementary Files

Supplementary Data 1

Reporting Summary

## Data Availability

All data are available in the main and supplementary files or available from authors upon reasonable request. Source Data underlying figures and tables are available as Supplementary Data 1. Repeats for gel experiments are shown in Supplementary Information.

## References

[1] Fehrentz T, Schönberger M, Trauner D (2011). Optochemical genetics. Angew. Chem. Int. Ed. Engl..

[2] Szobota S, Isacoff EY (2010). Optical control of neuronal activity. Annu. Rev. Biophys..

[3] Pless SA, Ahern CA (2013). Unnatural amino acids as probes of ligand-receptor interactions and their conformational consequences. Annu. Rev. Pharm. Toxicol..

[4] Huber T, Sakmar TP (2014). Chemical biology methods for investigating G protein-coupled receptor signaling. Chem. Biol..

[5] Manning G, Plowman GD, Hunter T, Sudarsanam S (2002). Evolution of protein kinase signaling from yeast to man. Trends Biochem. Sci..

[6] Kholodenko BN, Hancock JF, Kolch W (2006). Cell-signalling dynamics in time and space. Nat. Rev. Mol. Cell Biol..

[7] Kolch W, Halasz M, Granovskaya M, Kholodenko BN (2015). The dynamic control of signal transduction networks in cancer cells. Nat. Rev. Cancer.

[8] Huang EJ, Reichardt LF (2003). Trk receptors: roles in neuronal signal transduction. Annu. Rev. Biochem..

[9] Marlin MC, Li G (2015). Biogenesis and function of the NGF/TrkA signaling endosome. Int. Rev. Cell Mol. Biol..

[10] Hirose M, Kuroda Y, Murata E (2016). NGF/TrkA signaling as a therapeutic target for pain. Pain. Pract..

[11] Sofroniew MV, Howe CL, Mobley WC (2001). Nerve growth factor signaling, neuroprotection, and neural repair. Annu. Rev. Neurosci..

[12] Adriaenssens E (2008). Nerve growth factor is a potential therapeutic target in breast cancer. Cancer Res..

[13] Demir IE, Tieftrunk E, Schorn S, Friess H, Ceyhan GO (2016). Nerve growth factor & TrkA as novel therapeutic targets in cancer. Biochim. Biophys. Acta.

[14] Kaplan DR, Stephens RM (1994). Neurotrophin signal transduction by the Trk receptor. J. Neurobiol..

[15] Kaplan DR, Martin-Zanca D, Parada LF (1991). Tyrosine phosphorylation and tyrosine kinase activity of the trk proto-oncogene product induced by NGF. Nature.

[16] Klein R, Jing SQ, Nanduri V, O’Rourke E, Barbacid M (1991). The trk proto-oncogene encodes a receptor for nerve growth factor. Cell.

[17] Marchetti L (2013). Ligand signature in the membrane dynamics of single TrkA receptor molecules. J. Cell Sci..

[18] Biarc J, Chalkley RJ, Burlingame AL, Bradshaw RA (2013). Dissecting the roles of tyrosines 490 and 785 of TrkA protein in the induction of downstream protein phosphorylation using chimeric receptors. J. Biol. Chem..

[19] Obermeier A1 (1993). Tyrosine 785 is a major determinant of Trk-substrate interaction. EMBO J..

[20] Riccio A, Ahn S, Davenport CM, Blendy JA, Ginty DD (1999). Mediation by a CREB family transcription factor of NGF-dependent survival of sympathetic neurons. Science.

[21] Cunningham ME, Greene LA (1998). A function-structure model for NGF-activated TRK. EMBO J..

[22] Ye S (2010). Tracking G-protein-coupled receptor activation using genetically encoded infrared probes. Nature.

[23] Chen Y, Lu L, Ye S (2017). Genetic code expansion and optoproteomics. Yale J. Biol. Med..

[24] Liu CC, Schultz PG (2010). Adding new chemistries to the genetic code. Annu. Rev. Biochem..

[25] Chin JW (2017). Expanding and reprogramming the genetic code. Nature.

[26] Klippenstein V, Mony L, Paoletti P (2018). Probing ion channel structure and function using light-sensitive amino acids. Trends Biochem. Sci..

[27] Gautier A (2014). How to control proteins with light in living systems. Nat. Chem. Biol..

[28] Miller JC, Silverman SK, England PM, Dougherty DA, Lester HA (1998). Flash decaging of tyrosine sidechains in an ion channel. Neuron.

[29] Arbely E, Torres-Kolbus J, Deiters A, Chin JW (2012). Photocontrol of tyrosine phosphorylation in mammalian cells via genetic encoding of photocaged tyrosine. J. Am. Chem. Soc..

[30] Grunbeck A, Sakmar TP (2013). Probing G protein-coupled receptor-ligand interactions with targeted photoactivatable cross-linkers. Biochemistry.

[31] Chen Y (2017). Heritable expansion of the genetic code in mouse and zebrafish. Cell Res..

[32] Reddington S, Watson P, Rizkallah P, Tippmann E, Jones DD (2013). Genetically encoding phenyl azide chemistry: new uses and ideas for classical biochemistry. Biochem. Soc. Trans..

[33] Cunningham ME, Stephens RM, Kaplan DR, Greene LA (1997). Autophosphorylation of activation loop tyrosines regulates signaling by the TRK nerve growth factor receptor. J. Biol. Chem..

[34] Deisseroth K (2015). Optogenetics: 10 years of microbial opsins in neuroscience. Nat. Neurosci..

[35] Zhu S (2014). Genetically encoding a light switch in an ionotropic glutamate receptor reveals subunit-specific interfaces. Proc. Natl Acad. Sci. USA.

[36] Ye S (2008). Site-specific incorporation of keto amino acids into functional G protein-coupled receptors using unnatural amino acid mutagenesis. J. Biol. Chem..

[37] Meakin SO, MacDonald JI, Gryz EA, Kubu CJ, Verdi JM (1999). The signaling adapter FRS-2 competes with Shc for binding to the nerve growth factor receptor TrkA. A model for discriminating proliferation and differentiation. J. Biol. Chem..

[38] MacDonald JI, Gryz EA, Kubu CJ, Verdi JM, Meakin SO (2000). Direct binding of the signaling adaptor protein Grb2 to the activation loop tyrosines on the nerve growth factor receptor tyrosine kinase, TrkA. J. Biol. Chem..

[39] Tian M, Ye S (2016). Allosteric regulation in NMDA receptors revealed by the genetically encoded photo-cross-linkers. Sci. Rep..

[40] Rannversson H (2016). Genetically encoded photo-crosslinkers locate the high-affinity binding site of anti-depressant drugs in the human serotonin transporter. Nat. Commun..

[41] Ray-Saha S, Huber T, Sakmar TP (2014). Antibody epitopes on G protein-coupled receptors mapped with genetically encoded photoactivatable cross-linkers. Biochemistry.

[42] Coin I (2013). Genetically encoded chemical probes in cells reveal the binding path of urocortin-I to CRF class B GPCR. Cell.

[43] Poulsen MH, Poshtibana A, Klippensteina V, Ghisia V, Plesteda AJR (2019). Gating modules of the AMPA receptor pore domain revealed by unnatural amino acid mutagenesis. Proc. Natl Acad. Sci..

[44] Klippenstein V, Ghisi V, Wietstruk M, Plested AJR (2014). Photoinactivation of glutamate receptors by genetically encoded unnatural amino acids. J. Neurosci..

[45] Huber, T. & Sakmar T. P. Chemical biological methods for investigating G protein-coupled receptor signaling. *Chem. Biol.* **21**, 1224–1237 (2014). 10.1016/j.chembiol.2014.08.009 25237865

[46] Gautier A, Deiters A, Chin JW (2011). Light-activated kinases enable temporal dissection of signaling networks in living cells. J. Am. Chem. Soc..

[47] Luo J (2016). Genetically encoded optical activation of DNA recombination in human cells. Chem. Commun. (Camb.)..

[48] Duan L (2018). Optical activation of TrkA signaling. ACS Synth. Biol..

[49] Brown W, Liu J, Deiters A (2018). Genetic code expansion in animals. ACS Chem. Biol..

[50] Han S (2017). Expanding the genetic code of *Mus musculus*. Nat. Commun..

[51] Khamo JS, Krishnamurthy VV, Chen Q, Diao J, Zhang K (2019). Optogenetic delineation of receptor tyrosine kinase subcircuits in PC12 cell differentiation. Cell Chem. Biol..

[52] Chang KY (2014). Light-inducible receptor tyrosine kinases that regulate neurotrophin signalling. Nat. Commun..

[53] Maniatis, T., Sambrook, J., & Fritsch, E. F. *Molecular Cloning: A Laboratory Manual* (Cold Spring Harbor: Cold Spring Harbor Laboratory, 1989).

[54] Jullien J (2002). Trafficking of TrkA-green fluorescent protein chimerae during nerve growth factor-induced differentiation. J. Biol. Chem..

