# Supplementary material for: Photosensitive tyrosine analogues unravel site-dependent phosphorylation in TrkA initiated MAPK/ERK signaling
Source: Commun Biol. 2020 Nov 25;3:706. doi: 10.1038/s42003-020-01396-0 (PMC7689462; doi:10.1038/s42003-020-01396-0)
Supplement: Supplementary file 1 — Supplementary Information [file 42003_2020_1396_MOESM1_ESM.pdf]

Photosensitive tyrosine analogues unravel site-dependent phosphorylation in TrkA initiated MAPK/ERK signaling

Shu Zhao[1a#], Jia Shi[2#], Guohua Yu[1a], Dali Li[1b], Meng Wang[3], Chonggang Yuan[1a], Huihui Zhou[4], Amirabbas Parizadeh[5a], Zhenlin Li[5b], Min-Xin Guan[3], and Shixin Ye[5a,*]

[1]a) Shanghai Key Laboratory of Brain Functional Genomics, Ministry of Education, b) Shanghai Key Laboratory of Regulatory Biology, Institute of Biomedical Sciences and School of Life Sciences, East China Normal University (ECNU), Shanghai 200062, China.

[2]Department of Anesthesiology, State Key Laboratory of Cardiovascular Disease, Fuwai Hospital, National Center for Cardiovascular Diseases,Chinese Academy of Medical Sciences&Peking Union Medical College, Beijing 100037, China

[3]Division of Medical Genetics and Genomics, the Children's Hospital, Institute of Genetics, Zhejiang University School of Medicine, Hangzhou, Zhejiang, China.

[4]Brain Cognition and Brain Disease Institute (BCBDI), Shenzhen-Hong Kong Institute of Brain Science-Shenzhen Fundamental Research Institutions, Guangdong Provincial Key Laboratory of Brain Connectome and Behavior, CAS Key Laboratory of Brain Connectome and Manipulation, Shenzhen Institutes of Advanced Technology, Chinese Academy of Sciences, Shenzhen, 518055, China.

[5]a) UMR 7238 (LCQB unit), b) UMR 8256 (B2A unit), Centre National de la Recherche Scientifique (CNRS), Institut National de la Sante et de la Recherche Medicale (INSERM), Institute of Biology, Paris-Seine (IBPS), Sorbonne University, Paris 75005, France.

* Corresponding author:

Present affiliation: INSERM U1195 unit, University of Paris-Saclay, Le Kremlin Bicêtre, 94276, France

S.Y.: Tel.: (33) 01.44.27.60.57

Email: shixin.ye-lehmann@inserm.fr

# Equally contributing authors

Supplementary Figures 1-12

Supplementary Tables 1-2

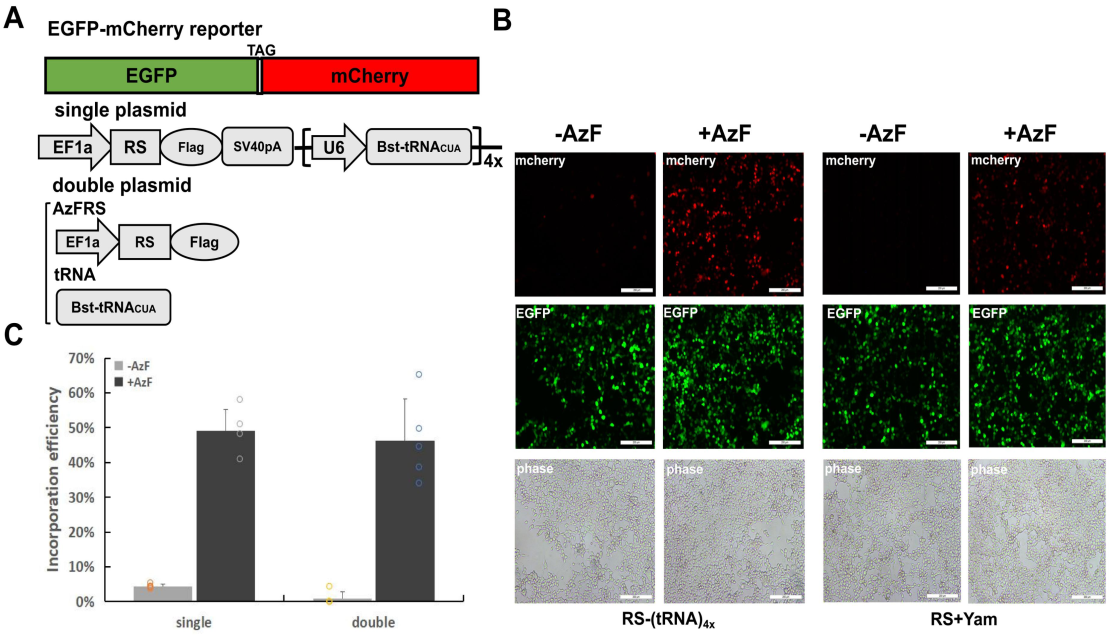

Supplementary Figure 1. Comparison of amber suppression yield through single and double plasmids transfection in HEK293T cells. **a.** Schematic diagram of EGFP-mCherry reporter. An amber mutation (TAG) was introduced between EGFP and mCherry. The single plasmid AzF-RS/tRNA$_{4X}$ encoding the orthogonal pair of AzF-RS and Bst-tRNA$_{CUA}$. The double plasmid constructs separately encoding AzF-RS and suppressor tRNA. **b.** Expression of EGFP-mCherry receptors in HEK293 cells. Fluorescent images of live cells co-transfected with either single plasmid AzFRS-tRNA$_{4X}$ or double plasmids separately encoding AzF-RS and the suppressor tRNA, cultured in the absence (left) or presence (right) of 1 mM AzF. Images were acquired on a Nikon Eclipse TE300 inverted microscope equipped with a Plan Fluor EL WD (objective 20x/0.45), (Scale bar: 200 μm). **c.** The expression of EGFP-mCherry AzF mutant in the absence (grey) or presence (dark) of AzF were described as average fluorescence intensity (%) in cells expressing UAA-incorporating mCherry divided by the average fluorescence intensity in cells expressing EGFP. Mean fluorescence intensity= indensity/area. n=6 independent experiments. Error bars show s.d.

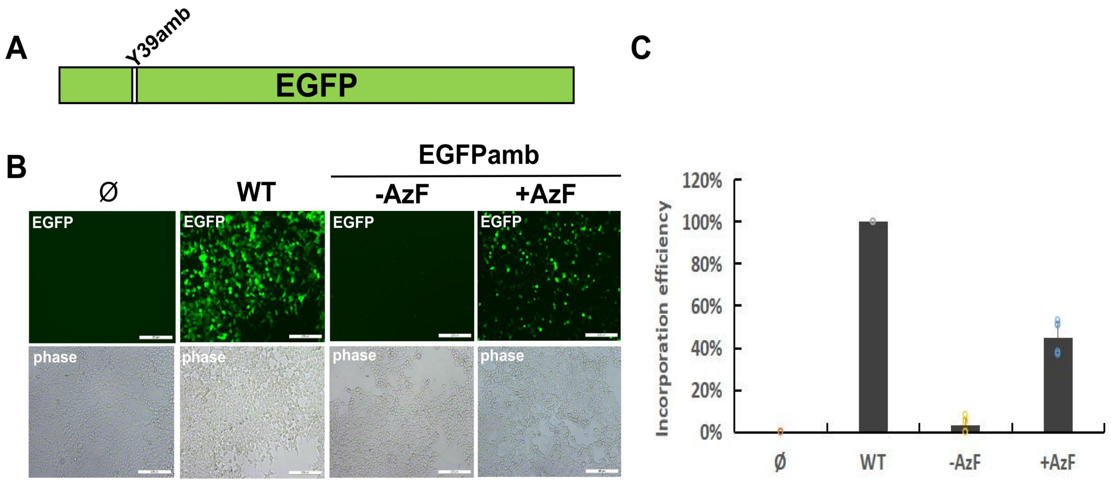

Supplementary Figure 2. Amber suppression in EGFP reporter. a. Schematic diagram of the constructed EGFP reporter with the amber mutation at the position Y39. **b.** Fluorescent images of non-transfected cells, cells transfected with wt-TrkA, cells co-transfected with EGFP reporter and RS/tRNA$_{4X}$, cultured in the presence or absence of 1 mM AzF. Eclipse TE300 inverted microscope equipped with a Plan Fluor EL WD (objective 20x/0.45)**,** (Scale bar: 200 µm) **c.** The expression of EGFP AzF mutant in the absence (grey) or presence (dark) of AzF were described as average fluorescence intensity (%) compared with WT TrkA. Mean fluorescence intensity= indensity/area. n=6 independent experiments. Error bars show s.d..

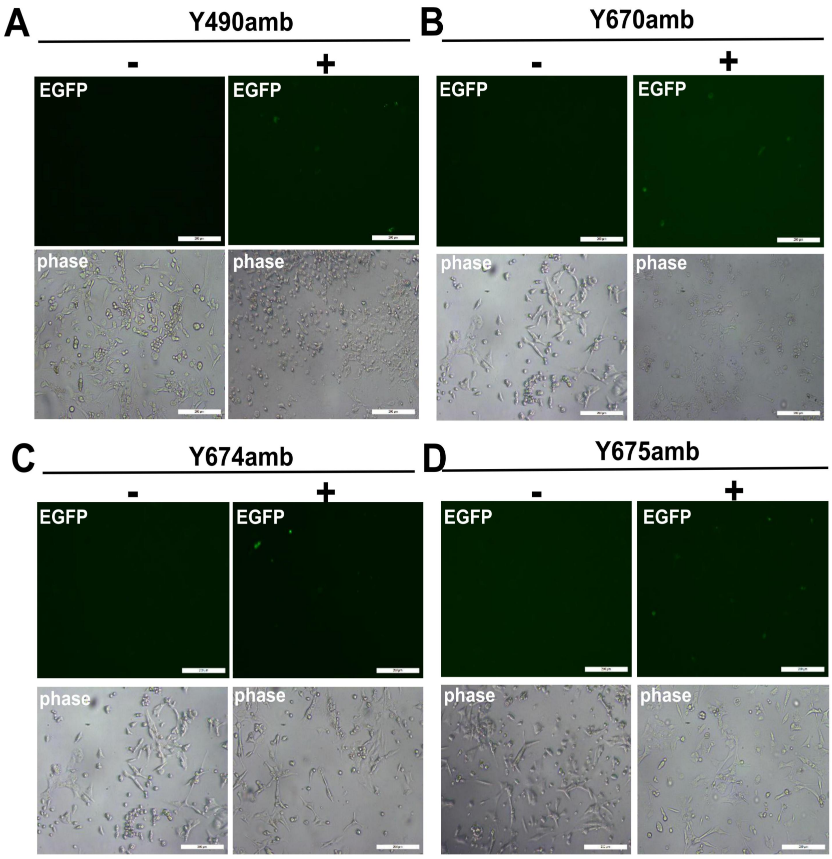

Supplementary Figure 3. Expression of TrkA-AzF mutants in PC12 cells. Fluorescent images of live cells co-transfected with RS/tRNA$_{4x}$ and TrkA-Y490amb (**a**), TrkA-Y670amb (**b**), TrkA-Y674amb (**c**), TrkA-Y675amb (**d**), in the presence (+) or absence (-) of 1 mM AzF. Scale bar: 200 µm. Eclipse TE300 inverted microscope equipped with a Plan Fluor EL WD (objective 20x/0.45), Scale bar: 200 µm.

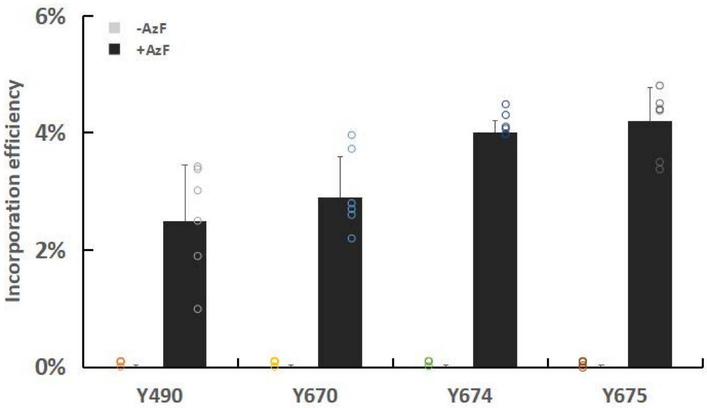

Supplementary Figure 4. Incorporation efficiency of TrkA-AzF mutants in PC12 cells. Average expression of TrkA-AzF mutants measured for indicated conditions in the absence (grey) or presence (dark) of AzF, mean fluorescence intensity= Indensity/area. n= 6 independent experiments. Error bars show s.d..

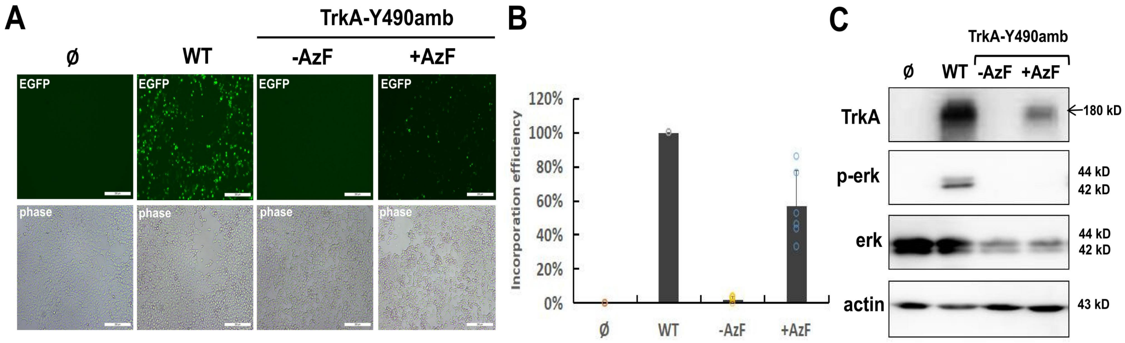

Supplementary Figure 5. Analysis of TrkA-Y490AzF mutant. a. Fluorescent images of non-transfected cells (Ø), cells transfected with wt-TrkA (WT), cells co-transfected with TrkA(490amb) and AzFRS-$tRNA_{4X}$, cultured in the absence (-AzF) or presence (+AzF) of 1 mM AzF. Scale bar: 200 µm. **b.** Average expression of TrkA measured for each condition, which was described as average fluorescence intensity (%) compared with WT TrkA. Mean fluorescence intensity= Intensity/area. n=6 independent experiments, Error bars show s.d.. **c**. Western blot analysis of HEK293T cells for TrkA, phosphorylated ERK (Thr202 and Tyr204), p42/44 MAPK (Erk1/2) for each condition corresponding to the imaging assay as indicated for **a** and **b**. Actin serves as a loading control.

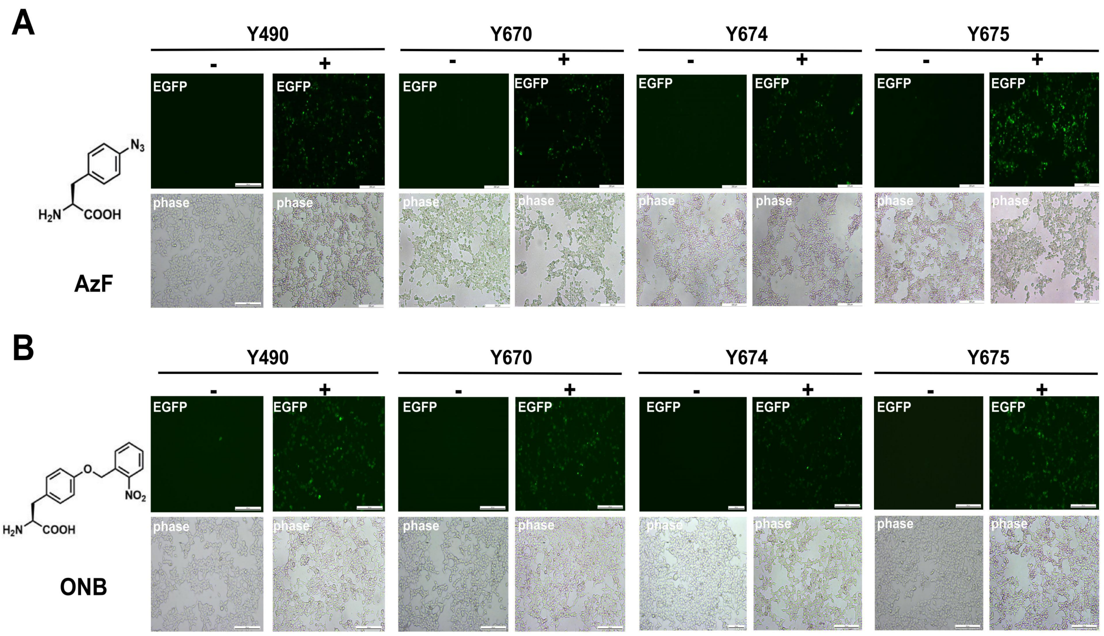

Supplementary Figure 6. Incorporation of AzF and ONB in tyrosine kinase domain sites. Fluorescent images of live cells co-transfected with RS/$tRNA_{4x}$ at one site out of tyrosine kinase domain Y490, and three different sites in tyrosine kinase domain TrkA-Y670, TrkA-Y674, TrkA-Y675, in the presence (+) or absence (-) of 1 mM AzF (**a**) and 1 mM ONB (**b**).

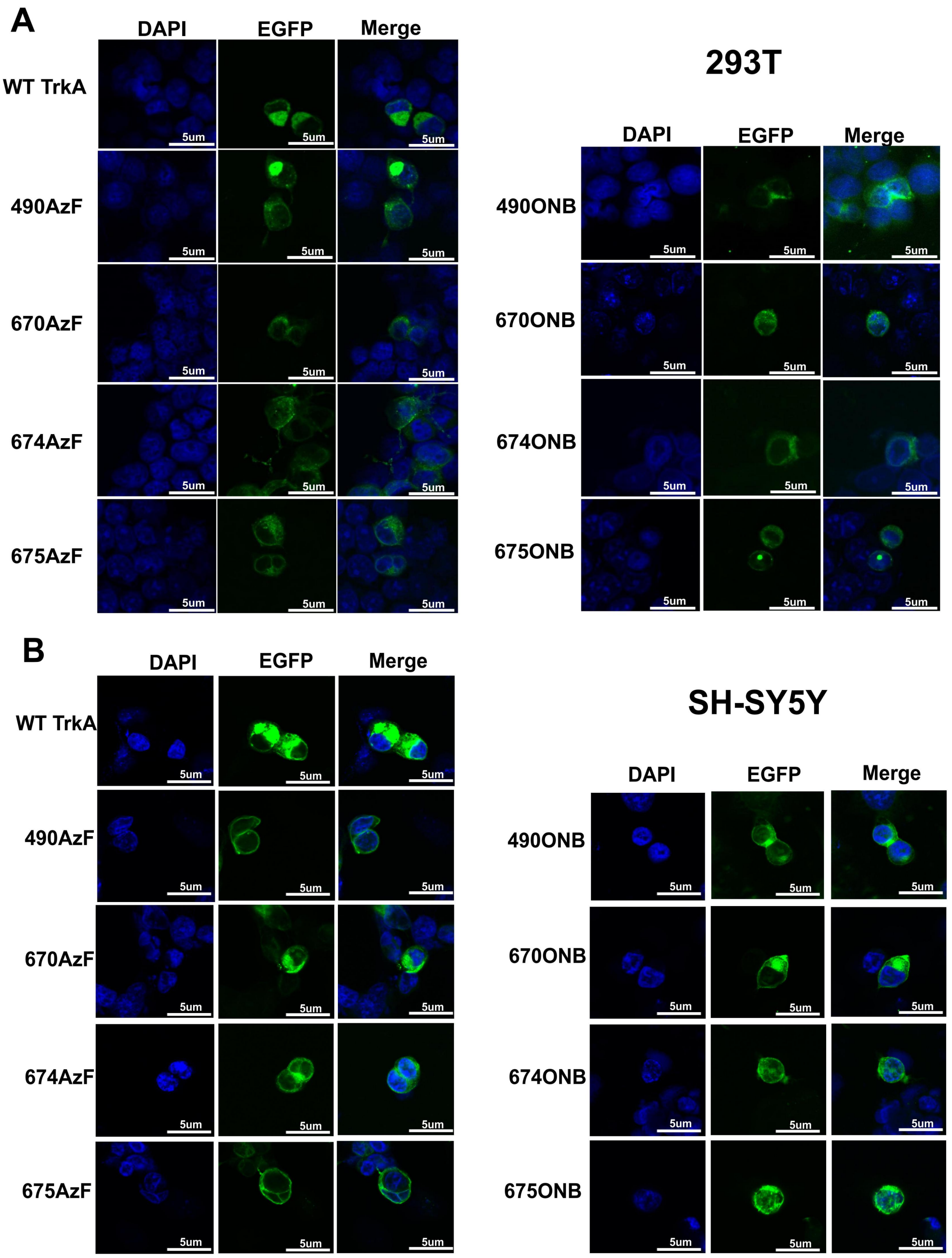

Supplementary Figure 7: Localization of TrkA constructs. Fluorescence images of WT TrkA receptors and TrkA-AzF or ONB mutants expressed in HEK293T (**a**) or SH-SY5Y cells (**b**). Blue, nuclei stained with 4', 6-diamidino-2-phenylindole (DAPI); green, EGFP tagged TrkA. Scale bars, 5 um.

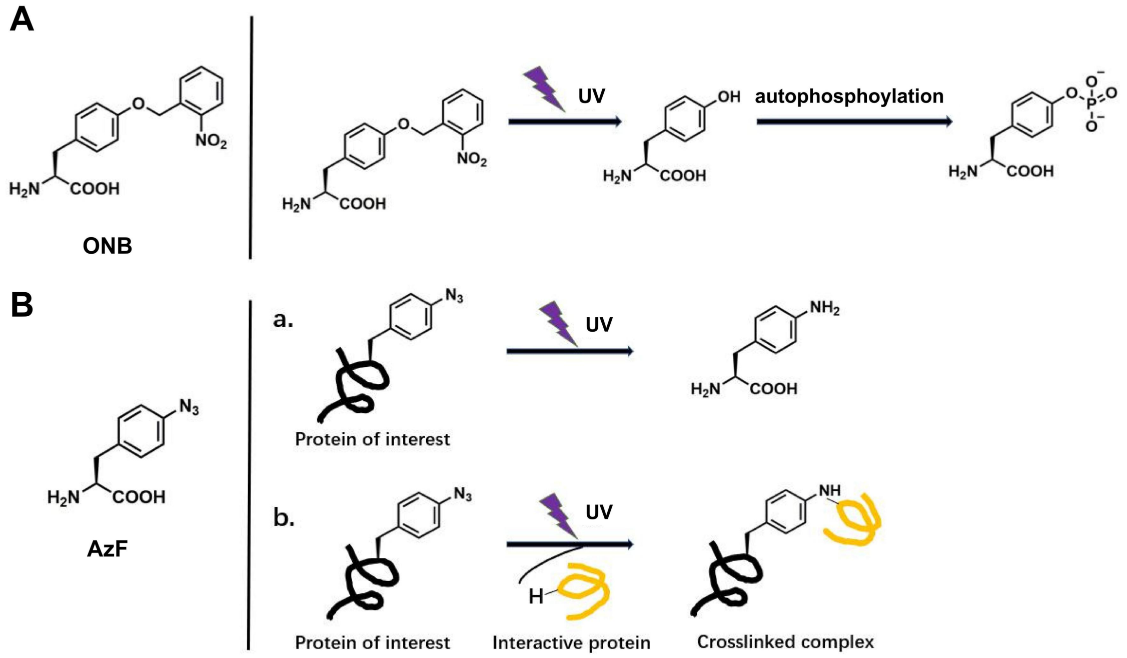

Supplementary Figure 8. AzF and ONB serve as light-switch to control the phosphorylation state. When exposed to UV light: ONB (**a**) releases a photolabile moiety from the side chain upon light activation, AzF (**b**) uses two paths either reduces to phenylamine (a) or forms covalent linkages with a nearby protein (b).

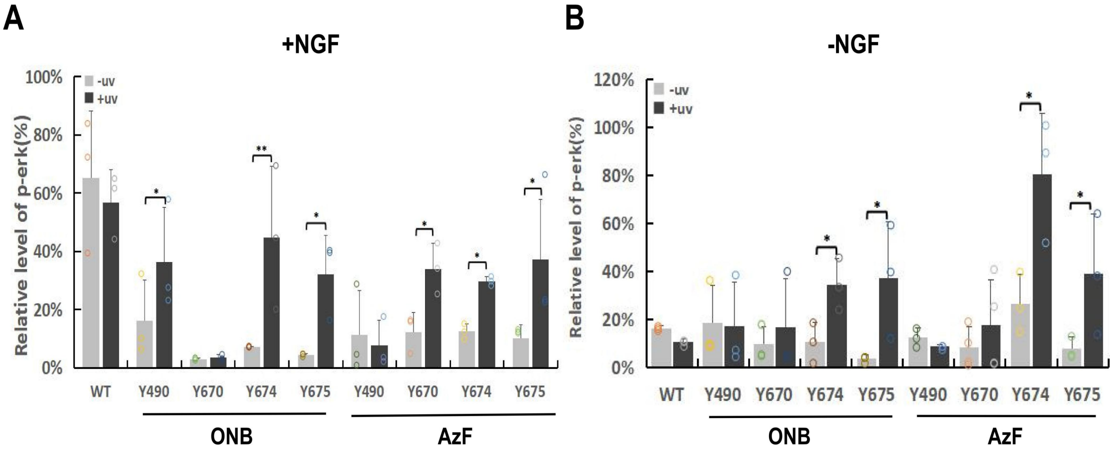

Supplementary Figure 9. Average level of p-erk in TrkA-ONB and TrkA-AzF after UV stimulation. The expression of phosphorylated ERK for four TrkA mutants in the absence (grey) or presence (dark) of UV. Cells were transfected with TrkA-ONB (**a**) and TrkA-AzF (**b**) respectively. Error bars show s.d.

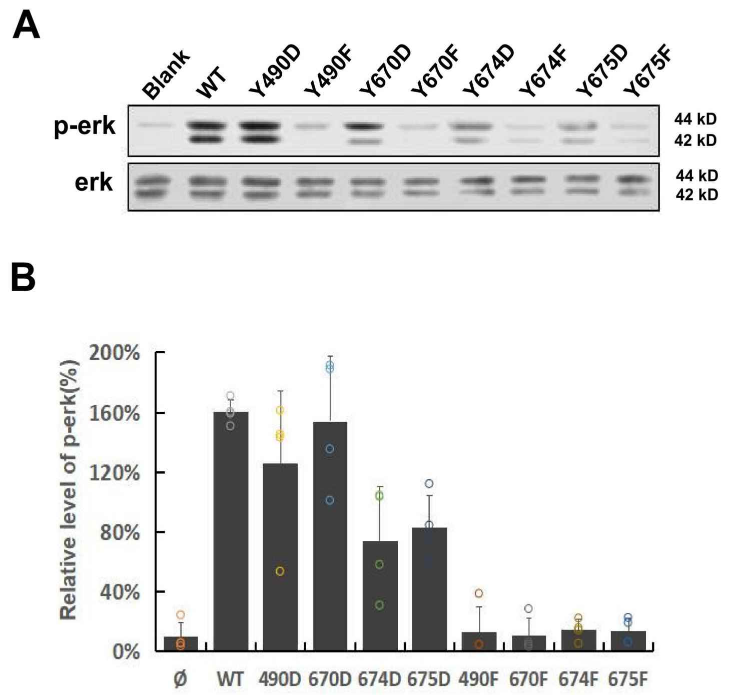

Supplementary Figure 10: The phosphorylation status of ERK with TrkA carrying phenylalanine (F) and aspartic acid (D) mutations. (**a**) Western blot analysis of HEK293T cells without transfection (Ø), wt-TrkA (wt) or expressing indicated mutants for phosphorylated ERK (Thr202 and Tyr204) in the presence of ligand NGF. (**b**) Relative level of p-erk/total erk. Error bars show s.d.

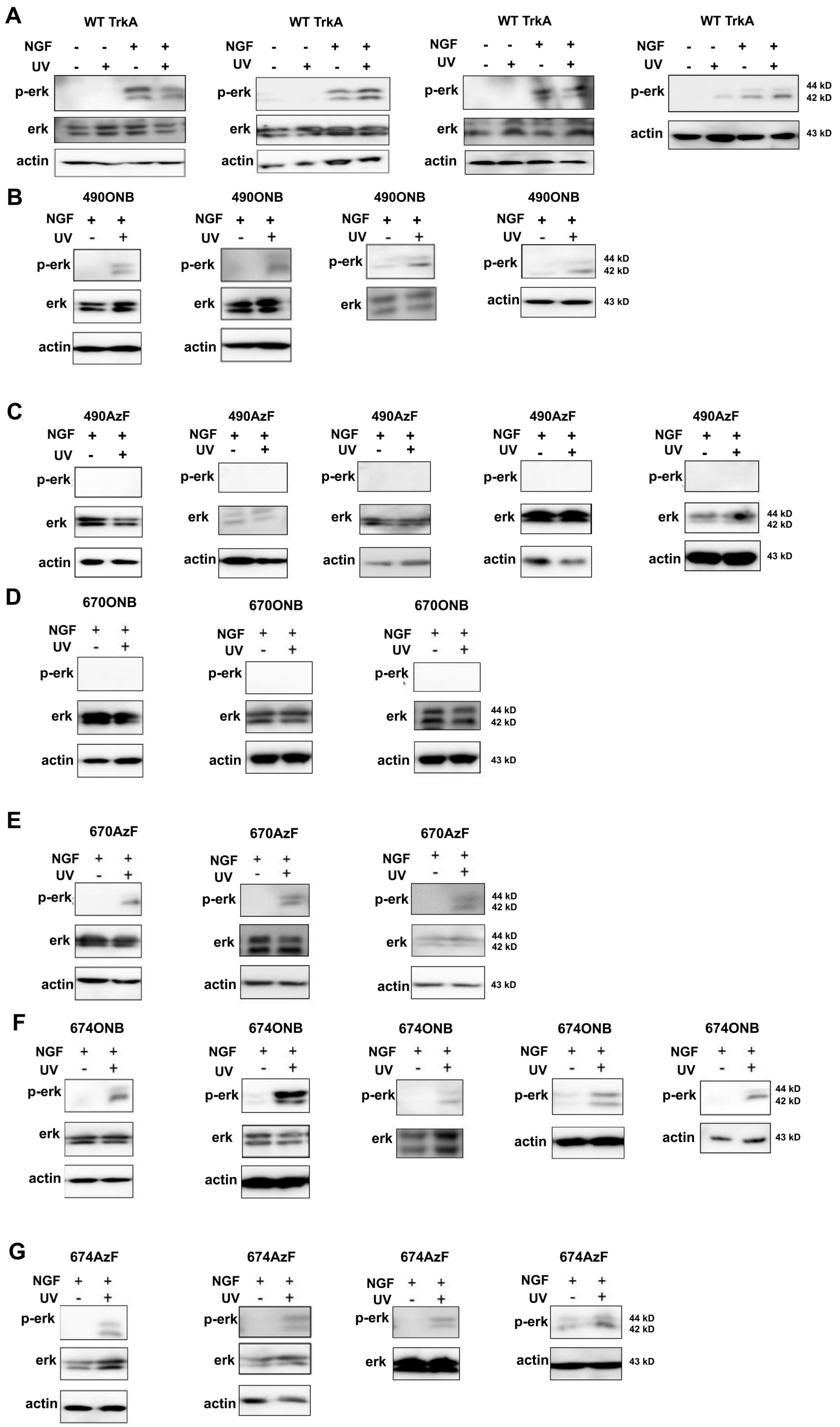

A
WT TrkA
NGF
UV
p-erk
erk
actin
44 kD
42 kD
43 kD
B
490ONB
C
490AzF
D
670ONB
E
670AzF
F
674ONB
G
674AzF

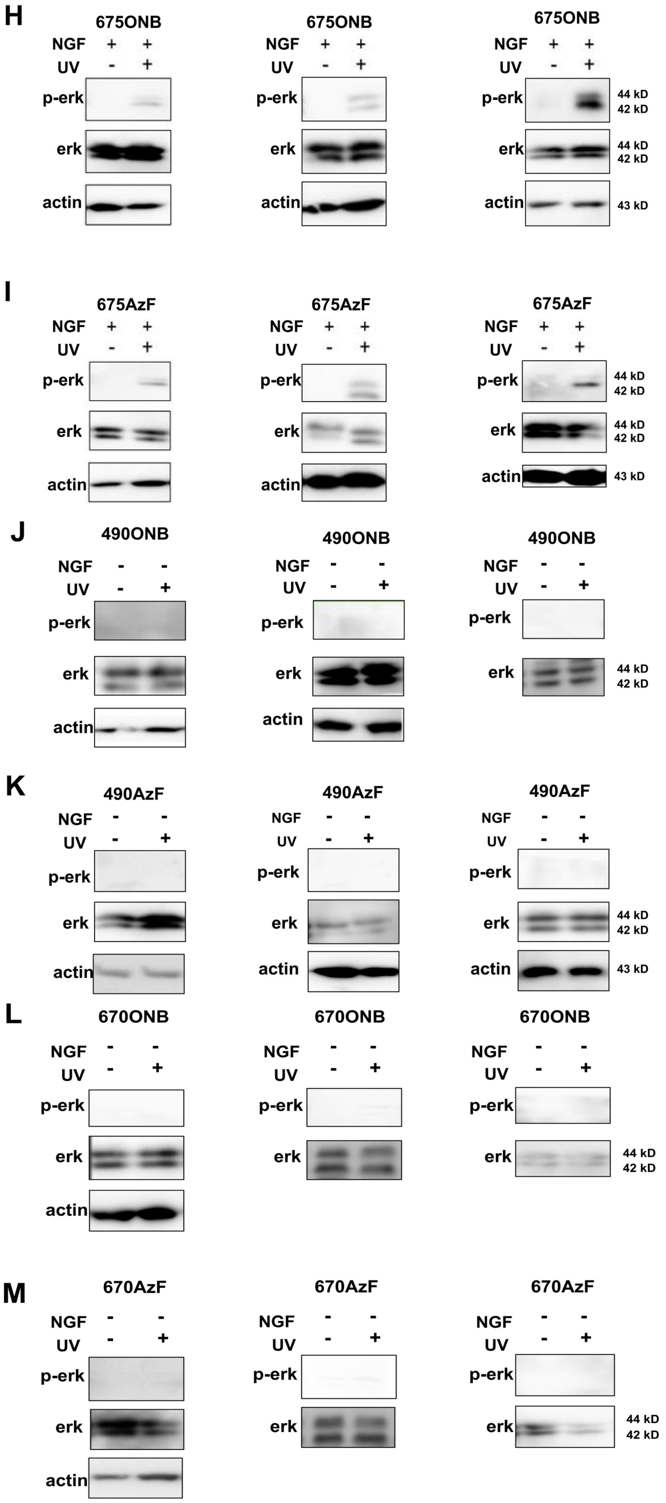
H
675ONB
NGF + +
UV - +
p-erk
erk
actin
44 kD
42 kD
43 kD
I
675AzF
NGF + +
UV - +
p-erk
erk
actin
44 kD
42 kD
43 kD
J
490ONB
NGF - -
UV - +
p-erk
erk
actin
44 kD
42 kD
K
490AzF
NGF - -
UV - +
p-erk
erk
actin
44 kD
42 kD
43 kD
L
670ONB
NGF - -
UV - +
p-erk
erk
actin
44 kD
42 kD
M
670AzF
NGF - -
UV - +
p-erk
erk
actin
44 kD
42 kD

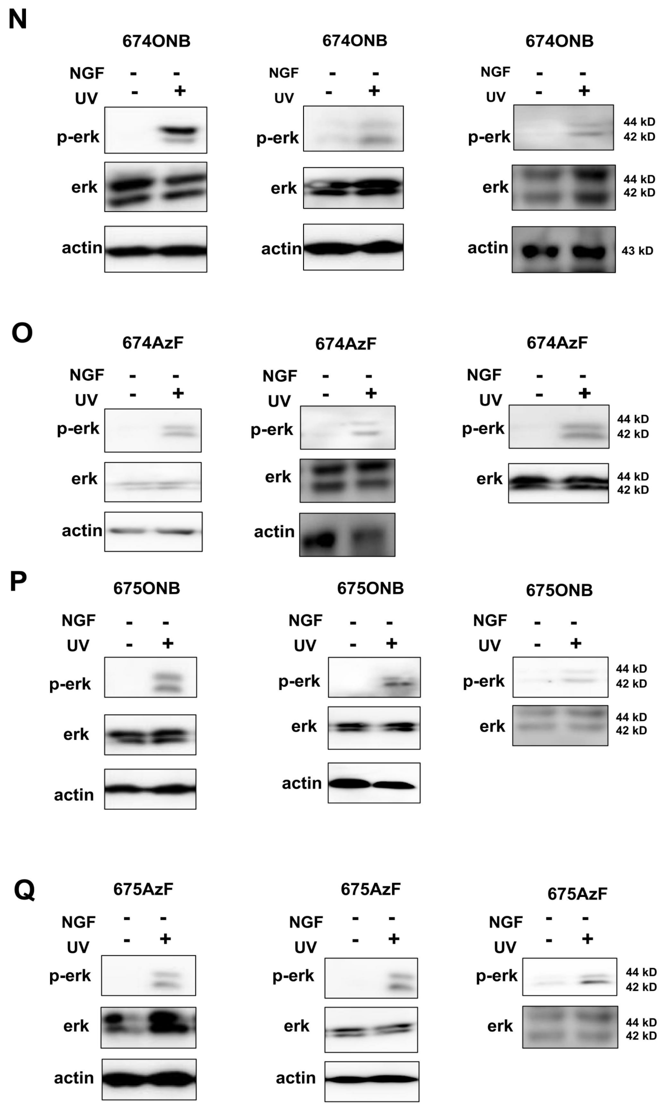

Supplementary Figure 11:Repeats for gel experiments. Western blot analysis of HEK293T cells expressing ONB mutants for phosphorylated ERK (Thr202 and Tyr204) in the presence of ligand NGF without (-) or with (+) UV light.

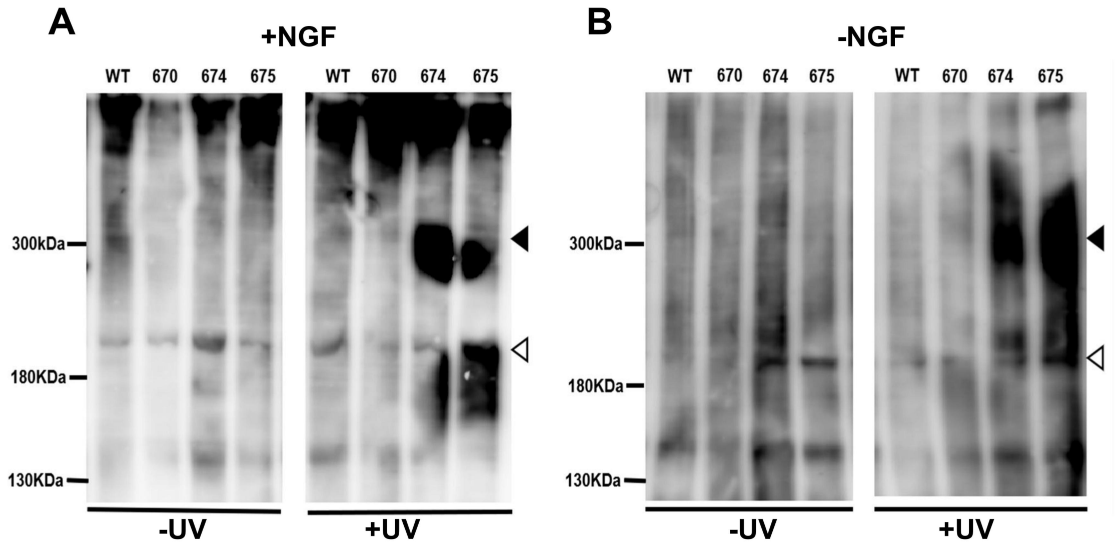

Supplementary Figure 12: Crosslinked proteins formation of TrkA-AzF mutants after UV treatment. (**a-b**) Immunoblots from HEK293T cells expressing WT TrkA receptors, TrkA-Y670AzF mutant, TrkA-Y674AzF mutant and TrkA-Y675AzF mutant. HEK293T cells were either treated with (+) or without (−) UV after (+/-) NGF treatment. UV photolysis was done with 365 nm UV cross-linker (40 w) for 5 min at 4°C . Samples were analyzed by anti-TrkA antibodies. TrkA monomer runs at ~180 kDa (indicated by a emtpy triangle) and TrkA-Y674AzF, TrkA-Y675AzF crosslinked proteins run at ~300 kDa with (**a**) or without (**b**) NGF treatment (indicated by solid triangle). WT and TrkA-Y670AzF have no obvious crosslinking band. Experiments were repeated 3 times.

Supplementary Table 1. The incorporation efficiency of TrkA-AzF and TrkA-ONB mutant.

Mutant sites	Uaa	Y490	Y670	Y674	Y675
Incorporation efficiency (%)	ONB	33.1±0.1	35.8±0.1	33.7±0.1	29.1±0.09
	AzF	52.6±0.07	37.4±0.09	37.1±0.01	33.4±0.06

Supplementary Table 2: Differentiation level of SH-SY5Y cells expressing TrkA AzF and ONB mutants.

A

Differentiation ratio(+NGF)（%）	AzF		ONB	
	-UV	+UV	-UV	+UV
TrkA-Y490	8.5±0.07	12.2±0.03	0.0±0.004	64.2±0.09
TrkA-Y670	2.6±0.04	31.9±0.1	0.0±0.004	1.7±0.04
TrkA-Y674	3.1±0.05	12.8±0.06	2.4±0.04	62.8±0.2
TrkA-Y675	2.6±0.04	22.2±0.06	7.5±0.06	53.3±0.1

B

Differentiation ratio (-NGF）	AzF		ONB	
	-UV	+UV	-UV	+UV
TrkA-Y490	0.7±0.8	0.2±0.07	0.4±0.1	1.3±0.7
TrkA-Y670	0.9±0.8	0.7±0.8	0.8±0.8	1.0±0.6
TrkA-Y674	0.4±0.2	5.9±0.9	0.5±0.4	3.9±0.6
TrkA-Y675	0.7±0.7	3.8±1.2	0.7±0.7	3.6±0.6
