# Supplementary material for: Photosensitive tyrosine analogues unravel site-dependent phosphorylation in TrkA initiated MAPK/ERK signaling
Source: Commun Biol. 2020 Nov 25;3:706. doi: 10.1038/s42003-020-01396-0 (PMC7689462; doi:10.1038/s42003-020-01396-0)
Supplement: Supplementary file 2 — Description of Additional Supplementary Files [file 42003_2020_1396_MOESM2_ESM.docx]

**Description of Additional Supplementary Files**

**File Name: Supplementary Data 1**

**Description**: The source data used to generate all graphs presented in the main figures (Figures 3, 6), and supplementary figures (Supplementary Figures 1, 2, 4, 5, 9, 10) of the result section.
